# A flow equilibrium of zinc in cells of *Cupriavidus metallidurans*

**DOI:** 10.1128/jb.00080-24

**Published:** 2024-04-25

**Authors:** Dietrich H. Nies, Grit Schleuder, Diana Galea, Martin Herzberg

**Affiliations:** 1 Martin-Luther-University Halle-Wittenberg, Institute for Biology/Microbiology, Halle (Saale), Germany; 2 Department of Analytical Chemistry, Helmholtz Centre for Environmental Research – UFZ, Leipzig, Germany; Queen Mary University of London, London, United Kingdom

**Keywords:** *Cupriavidus metallidurans*, zinc, zinc transport

## Abstract

The hypothesis was tested that a kinetical flow equilibrium of uptake and efflux reactions is responsible for balancing the cellular zinc content. The experiments were done with the metal-resistant bacterium *Cupriavidus metallidurans*. In pulse-chase experiments, the cells were loaded with radioactive ^65^Zn and chased with the 100-fold concentration of non-radioactive zinc chloride. In parallel, the cells were loaded with isotope-enriched stable ^67^Zn and chased with non-enriched zinc to differentiate between zinc pools in the cell. The experiments demonstrated the existence of a kinetical flow equilibrium, resulting in a constant turnover of cell-bound zinc ions. The absence of the metal-binding cytoplasmic components, polyphosphate and glutathione, metal uptake, and metal efflux systems influenced the flow equilibrium. The experiments also revealed that not all zinc uptake and efflux systems are known in *C. metallidurans*. Cultivation of the cells under zinc-replete, zinc-, and zinc-magnesium-starvation conditions influenced zinc import and export rates. Here, magnesium starvation had a stronger influence compared to zinc starvation. Other metal cations, especially cobalt, affected the cellular zinc pools and zinc export during the chase reaction. In summary, the experiments with ^65^Zn and ^67^Zn demonstrated a constant turnover of cell-bound zinc. This indicated that simultaneously occurring import and export reactions in combination with cytoplasmic metal-binding components resulted in a kinetical flow equilibrium that was responsible for the adjustment of the cellular zinc content.

**IMPORTANCE:**

Understanding the biochemical action of a single enzyme or transport protein is the pre-requisite to obtain insight into its cellular function but this is only one half of the coin. The other side concerns the question of how central metabolic functions of a cell emerge from the interplay of different proteins and other macromolecules. This paper demonstrates that a flow equilibrium of zinc uptake and efflux reactions is at the core of cellular zinc homeostasis and identifies the most important contributors to this flow equilibrium: the uptake and efflux systems and metal-binding components of the cytoplasm.

## INTRODUCTION

No organisms are known that can survive without zinc ions, which are essential cofactors in many enzymes (1), for example, RNA polymerase (2–4). Nevertheless, zinc ions are toxic at higher concentrations. Zinc homeostasis requires a walk on a fine line between zinc starvation on the one hand and zinc toxicity on the other hand (5).

**Fig 1:**
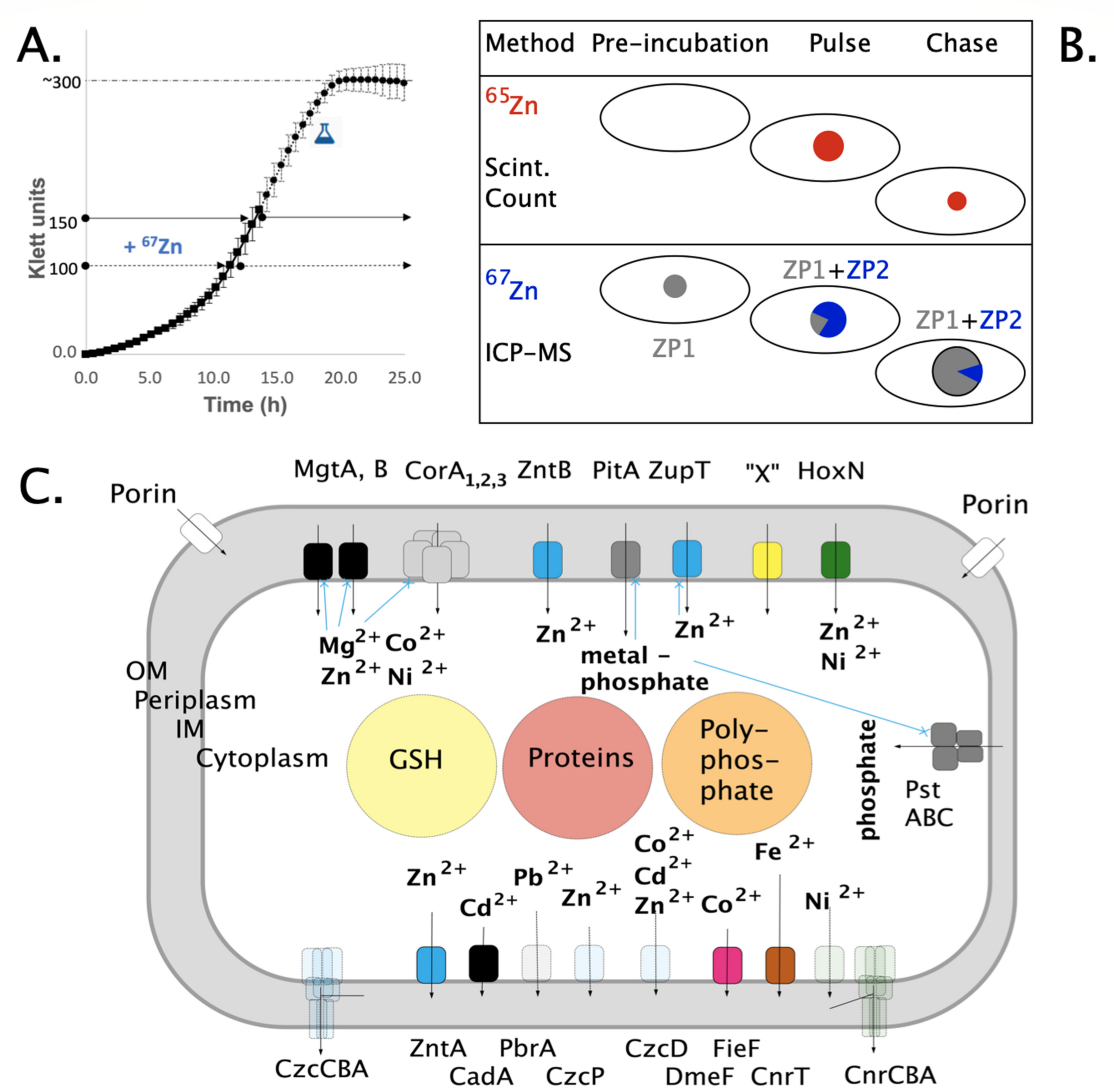
Schematic diagram of the methods used and the transport systems involved. Panel A. Growth curve of *C. metallidurans*. For the establishment of the method using stable ^67^Zn, the metal was added at 100 Klett units, the cells were harvested and analyzed by inductively coupled plasma mass spectrometry (ICP-MS) at 150 Klett units. Panel B. Cells harvested at 150 Klett units were used for pulse-chase studies with radioactive ^65^Zn (red) and in parallel with isotope-enriched ^67^Zn (blue). The ellipsoids are the cells, the circles inside the zinc pools. Radioactive ^65^Zn is the only zinc that can be measured with the scintillation counter. With stable isotope-enriched zinc and ICP-MS, a pool ZP1 (gray) with the natural isotope composition could be discriminated from a pool with a higher percentage of ^67^Zn ZP2 (blue). Panel C. Transition metal cations and Mg(II) cross the outer membrane (OM) into the periplasm (gray) and further into the cytoplasm by uptake systems (on top). They can be bound inside the cells by glutathione, proteins, and polyphosphate (big circles). They may be exported from the cytoplasm to the periplasm by efflux systems (bottom row) and further on the outside by RND-driven transenvelope efflux systems (corners). Plasmid-encoded systems dashed, regulatory events concerning uptake systems (5) in blue.

*Cupriavidus metallidurans* strain CH34 is a beta-proteobacterium adapted to high concentrations of zinc and other transition metal cations (6) but is surprisingly also able to manage zinc starvation conditions (7–9). It survives in environments such as zinc deserts and auriferous soils (10–13). Its genome is composed of a chromosome, a chromid, and two large plasmids, which all contain metal resistance determinants that were acquired during the evolution of this bacterium by horizontal gene transfer (6, 14–16). The high level of zinc resistance is mediated by the *czc* (cobalt-zinc-cadmium) resistance determinant on plasmid pMOL30, which encodes two inner membrane efflux systems and the transenvelope efflux complex CzcCBA [Fig. 1 (5, 17)]. Without plasmid pMOL30 or *czc* on this plasmid, zinc resistance of *C. metallidurans* is on a similar level as that of *Escherichia coli* as has been determined, for instance, in the plasmid-free strain *C. metallidurans* AE104 (18, 19).

**Fig 2:**
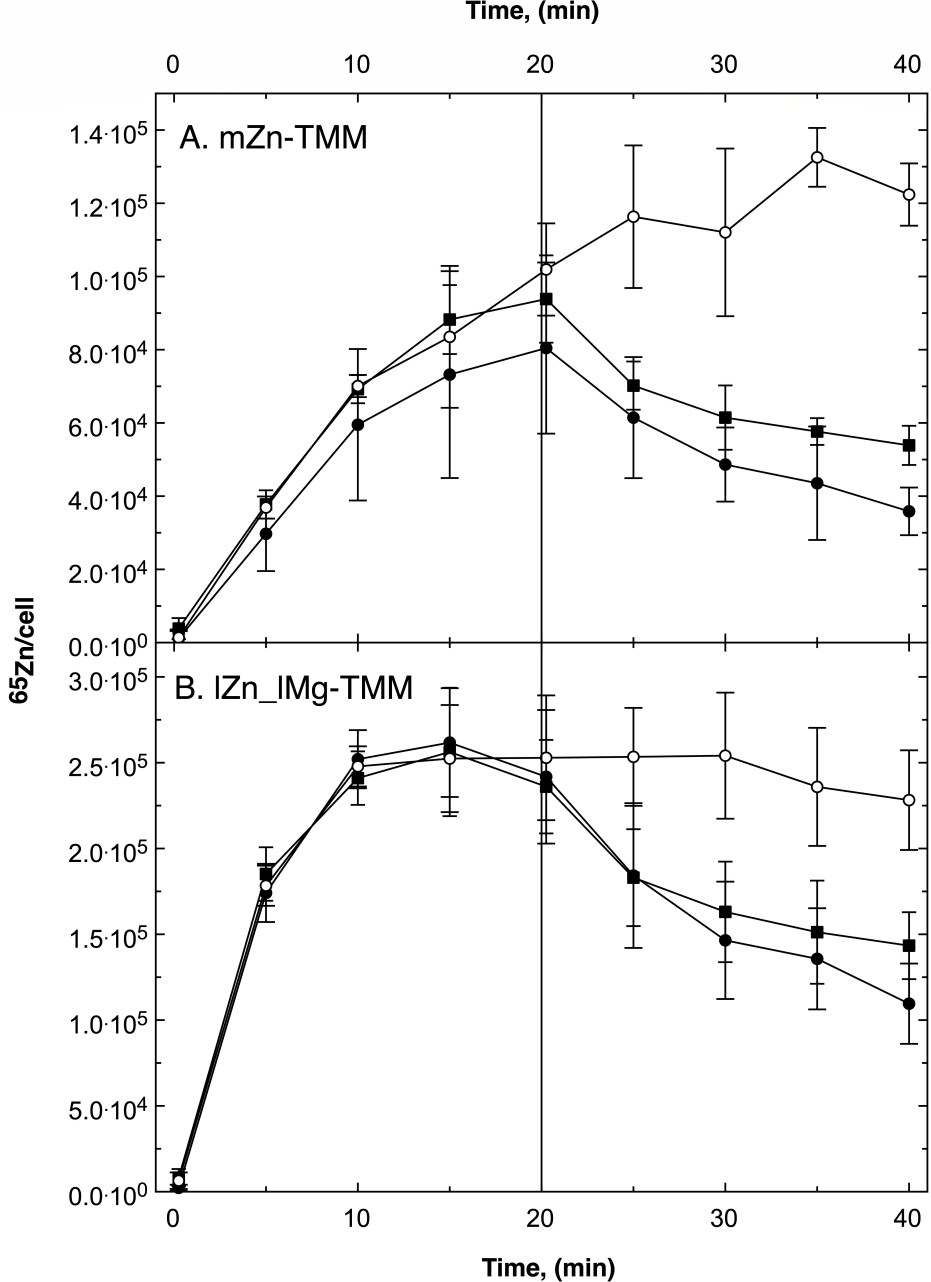
Pulse-chase experiment with *C. metallidurans* strain AE104 and zinc. The cells were cultivated in TMM containing 200 nM Zn(II) (mZn-TMM, Panel A) or in a low metal medium without added zinc and 100 µM Mg(II) instead of 1 mM (lZn_lMg-TMM, Panel B). A preculture of the same medium was used. At turbidity of 150 Klett units, the cells were harvested by centrifugation, suspended in an equal volume of 10 mM TrisHCl (pH 7.0), and stored on ice until needed but no longer than a few hours. To 6 mL of this cell suspension, 2 g/L Na gluconate was added before the experiment. The uptake reaction (pulse) was started by the addition of 12 µL ^65^Zn (500 µM, 12 µCi, 1 µM final concentration). The cells were incubated with shaking at 30°C. Samples of 500 µL were collected by filtration (0.2 µm pore size), washed twice with 5 mL ice-cold wash solution (50 mM Tris-HCl pH 7.0, 50 mM EDTA), and radioactivity was measured in a scintillation counter. After 20 min (bar), non-radioactive Zn(II) was added (chase) to a final concentration of 100 µM (closed circles), 1 mM (closed squares) or not (open circles) and sampling was continued. Three biological repeats and deviation bars are given.

Strain AE104 contains on its chromosome and chromid two inner membrane efflux pumps ZntA and CadA that belong to the P_IB2_-type ATPases, and the two CDF proteins DmeF and FieF of the cation diffusion family of exporters [Fig. 1, (5)]. These proteins mediate in strain AE104 an IC_50_ of zinc of about 1 mM compared to 3.4 mM in pMOL30-containing strains (19). Deletion of the genes for the two P_IB2_-type ATPases results in a decrease of the IC_50_ to 7.7 µM and this value declined only slightly further when *dmeF* and *fieF* were additionally eliminated (19) so that ZntA and CadA were responsible for zinc efflux in strain AE104. While *zntA* was similarly up-regulated by Zn(II) in the presence or absence of CadA, *cadA* was only up-regulated by Zn(II) in the absence of ZntA (20). ZntA is the main inner membrane efflux system for Zn(II) in strain AE104 and CadA with respect to zinc and especially cadmium a backup system. Expression of both genes for these P_IB2_-type ATPases was regulated by a MerR-type regulator, ZntR and CadR, respectively (7) so that ZntR and ZntA maintain zinc homeostasis in strain AE104 at higher zinc concentrations.

Zinc ions were imported into TMM-grown AE104 cells with a *K*_m_ of 137 ± 87 µM and a v_max_ of 3.7 ± 2.1 µmol min^−1^ g^−1^ dry weight. Uptake was competitively inhibited by Mg(II) and the import rate increased sevenfold when the cells were cultivated in TMM (Tris-buffered mineral salts medium) with 100 µM Mg(II) instead of 1 mM magnesium in the standard TMM (21). Assuming a dry mass of 615 fg per cell as calculated from the cellular dimensions of *C. metallidurans* (22), the v_max_ of 3.7 ± 2.1 µmol min^−1^ g^−1^ dry weight would mean an import of 22,800 ± 13,000 Zn(II) s^−1^ cell^−1^. Following Michaelis-Menten kinetics, this would mean an initial import rate at 1 µM Zn(II) of 166 ± 94 Zn(II) s^−1^ cell^−1^.

Responsible for zinc import into *C. metallidurans* cells are at least nine import systems (Fig. 1). The ZIP protein [TC 2.A.5; Transporter Classification Database; (23, 24)] ZupT is up-regulated under conditions of zinc starvation (25). Expression of *zupT* is controlled by the zinc uptake regulator Zur, as in many bacteria (8, 9, 26–29). Four MIT (TC 1.A.35) proteins, CorA_1_, CorA_2_, CorA_3_, and ZntB, are not regulated by zinc but CorA_1_ is by magnesium (25) and the metal-phosphate importer PitA of the PiT family (protein inorganic transport family, TC 2.A.20) by phosphate. The HoxN NiCoT (Nickel-cobalt transporter family, TC 2.A.52) protein should be mainly a Ni(II) importer and the two P-type ATPases (TC 3.A.3) MgtA and MgtB Mg(II)/Ca(II) importers (25, 30). *C. metallidurans* does not contain an ABC-type (TC 3.A.1) zinc importer such as ZnuABC from *E. coli* (25, 27, 31, 32) so ZupT is an important zinc importer in this bacterium.

Deletion of *zupT* leads to a pleiotropic phenotype, for instance lacking incorporation of zinc into the beta-prime subunit RpoC of the RNA polymerase, although the cells were very well able to import zinc ions (33). Deletion of all seven known secondary zinc importers ZupT, CorA_1_, CorA_2_, CorA_2_, ZntB, PitA, and HoxN (Fig. 1) in mutant strain ∆7 did not abolish zinc import but reduced fitness and metal resistance of the cells (34). Not even the additional deletion of *mgtA* and *mgtB* in the mutant strain ∆9 prevented zinc uptake but decreased the fitness of the cell even more (30, 34) so that at least one additional zinc import pathway exists in *C. metallidurans* (Fig. 1C, upper row, yellow “X”).

The uptake and efflux systems of *C. metallidurans* strain AE104 may be responsible for the cellular zinc content as the result of a kinetical flow equilibrium with the activity of the individual transport systems regulated by gene expression, flux control, and other processes (5, 35, 36). Such a flow equilibrium would be a futile cycle that is required to constantly adjust the composition of the cellular metal pool and the concentration of each metal, all in cooperation with the metal-binding capacity of the cytoplasm. This process would be at the core of the multiple metal homeostasis of the cell; however, its very existence has not been demonstrated.

If such a kinetical flow equilibrium exists, metal ions should be continuously imported and exported into and out of the cell. Cells incubated with radioactive ^65^Zn should import the metal cation. Subsequent incubation with a higher concentration of non-radioactive Zn(II) (pulse-chase experiment) should not only decrease the uptake rate by dilution of the radioactive substrate but should result in a decrease of the ^65^Zn content of the cells (Fig. 1B). The flow equilibrium should be altered in the absence of uptake and efflux systems. Metal starvation conditions, leading to up- or down-regulation of transport systems, are supposed to change the flow equilibrium too. Moreover, different media should influence the supply of zinc ions and other metals to metal-requiring enzymes. To analyze the zinc pools in *C. metallidurans*, additionally, the stable zinc isotope ^67^Zn was used. This allows to accompany the ^65^Zn with parallel ^67^Zn pulse-chase experiments to discriminate between zinc that was originally present in the cells with a natural isotope composition, isotope-enriched zinc that is accumulated by the cells during the pulse phase, and the change of both zinc pools during the chase (Fig. 1B). In this publication, we show that a flow equilibrium of zinc import and export indeed exists and that this flow equilibrium impacts with the zinc pools of the *C. metallidurans* cell.

## RESULTS

### Experimental approach and outline

To investigate the kinetical flow equilibrium of zinc in the plasmid-free strain *C. metallidurans* AE104 and the influence of this flow equilibrium on the zinc pools in the cell, radioactive ^65^Zn was used to measure the zinc uptake and efflux reactions (Fig. 1). This work was mainly carried out with the plasmid-free *C. metallidurans* strain AE104 and its mutants (Table S1) because deletion of *zupT* for this important zinc importer is only possible in the absence of the plasmids (18, 33). For the pulse-chase experiments, the cells were loaded for 20 min with 1 µM ^65^Zn and subsequently chased with 100 µM non-radioactive zinc (Fig. 1 and 2). Controls were not chased. Experiments with other metals or 1 mM zinc were also performed.

In parallel, stable enriched ^67^Zn was employed to determine changes in the cellular zinc pool. Here, the cells were loaded with 1 µM ^67^Zn and chased with 100 µM zinc ions that were not enriched in ^67^Zn. After harvest, the cellular zinc content was determined using inductively coupled plasma-mass spectrometry (ICP-MS). The natural isotope composition of stable zinc is 48.6% ^64^Zn, 27.9% ^66^Zn, 4.1% ^67^Zn, and 0.6% ^70^Zn (37). The ICP-MS procedure measures the abundance of every isotope and calculates from each of these results the amount of total zinc by dividing it by the percentage of the respective isotope in the natural isotope composition. Using a solution with 94% ^67^Zn leads to an over-calculation of the total amount of zinc in the ^67^Zn compared to the ^64^Zn channel. From the ^64^Zn- and over-calculated ^67^Zn-values, the amount of zinc in the zinc pool ZP1 with the natural isotope composition can be calculated, and that of ZP2, which represents the additional zinc pool stemming from the ^67^Zn-isotope-enriched solution. That way, two zinc pools can be measured with ^67^Zn. By contrast, the method using radioactive ^65^Zn determines only this isotope (Fig. 1).

After the kinetical flow equilibrium had been analyzed in *C. metallidurans* AE104, other transition metal cations were also used for the chase period. The influence of metal uptake, efflux, and metal-binding cellular components on the zinc pools and the flow equilibrium was characterized using mutants of *C. metallidurans* AE104 (Table S1). Finally, *C. metallidurans* CH34 wild type was included, which possessed all the plasmid-encoded metal transport systems (Fig. 1).

### Proof of concept for the methodology used

All bacteria were cultivated in three derivatives of the Tris-buffered mineral salts medium TMM (18) with gluconate as the carbon source to the exponential phase of growth (Fig. 1). In moderate zinc medium mZn, the zinc content was adjusted to 200 nM. The second medium was a low zinc medium lZn that was TMM without the trace element solution SL6 (38) and contained 35.2 ± 30.4 nM zinc. It was used to investigate the influence of zinc starvation on the flow equilibrium of zinc. The third medium did not contain SL6 and also a lowered magnesium concentration of 0.1 mM instead of 1 mM so that magnesium starvation was added on top of the zinc starvation condition.

To obtain a proof of concept for the usage of ^67^Zn to measure different zinc pools in the cell, the cells were cultivated in these three media with Zn(II) present in the natural isotope composition to the mid-exponential phase of growth at 150 Klett units (Fig. 1) and harvested. The zinc content was determined by ICP-MS (Table S2). The cellular zinc content was at the expected and published level of 100,000 Zn per cell in the presence of 200 nM Zn(II) in mZn-TMM medium and lower zinc contents under zinc starvation conditions. The total zinc content of the cells as determined by the ICP-MS method was a half-exponential function of the zinc content of the growth medium (Fig. S1A). No zinc appeared in the zinc pool ZP2, which was an indicator of imported zinc from the ^67^Zn-isotope-enriched solution. All zinc resided in the ZP1, which represented zinc in the natural isotope composition.

In the next step, 1, 10, or 100 µM of ^67^Zn-isotope-enriched or not -enriched solution was added at a turbidity of 100 Klett units and the cells were harvested at 150 Klett units, which was still in the mid-exponential phase of growth (Fig. 1A). With increasing zinc concentration, the total zinc content ZP1 + ZP2 increased to very similar levels in cells cultivated in the three different media, in the absence or presence of ^67^Zn-isotope-enriched solution (Table S2). But zinc appeared in ZP2 only when ^67^Zn-isotope-enriched solution was used. Moreover, the zinc content in ZP2 increased at the cost of a decreasing zinc content in ZP1. When 1 to 100 µM zinc was added to the cells, zinc that was already residing in the cells in ZP1 with the natural isotope composition was exchanged against incoming isotope-enriched ^67^Zn that went into ZP2 (Table S2). There was no influence of the usage of ^67^Zn-isotope-enriched zinc solution on the content of other metals in *C. metallidurans* (Table S3).

A ^67^Zn-isotope-enriched zinc solution could be used to discriminate between incoming zinc (ZP2, isotope-enriched) and already residing zinc (ZP1, natural isotope composition). This method strictly depended on the usage of the ^67^Zn-isotope-enriched zinc solution and did not influence the cellular content of other metals. Moreover, first evidence was obtained that cell-bound zinc was exchanged for incoming zinc.

Compared to the zinc content determined by the ICP-MS method, the content of radioactive ^65^Zn increased with a double-logarithmical function (Fig. S1B). This was due to different procedures used to harvest the cells, rapid filtration and quick washing with ^65^Zn, centrifugation, and washing for the determination by ICP-MS. At 1 µM and incubation times of 2 h and below, the ^65^Zn data were in the same range as ZP2 determined with ^67^Zn so both methods gave compatible results when the cells were loaded for 20 min with 1 µM Zn(II), between 70,000 and 100,000 zinc per cell.

In summary, at zinc concentrations up to 1 µM, determination of the cellular zinc content using radioactive zinc and by ICP-MS gave similar results. At higher zinc concentrations, labeling with radioactive zinc yielded a higher cellular zinc content than centrifugation followed by ICP-MS measurement, indicating that the ^65^Zn method revealed a more loosely bound zinc pool than the ICP-MS method.

### *C. metallidurans* strain AE104 as a reference point

Pulse-chase experiments with the plasmid-free *C. metallidurans* strain AE104 were performed in moderate zinc medium mZn-TMM adjusted to 200 nM Zn(II), in low zinc lZn and low zinc, low magnesium lZn_lMg medium, yielding zinc-replete, zinc-starved, and metal-starved cells, respectively. After loading with 1 µM of radioactive ^65^Zn, the cells were chased with 1 mM or 100 µM non-radioactive zinc (Fig. 2), 100 µM of other metal cations, or the metal chelator EDTA (ethylenediaminetetraacetate) (Fig. 2; Fig. S2). From these data, the initial uptake v_up_(0), initial efflux rate v_eff_(0) at the beginning of the chase, zinc contents after 20 min C_20_, extrapolated zinc equilibrium content after the uptake phase C_max_, calculated zinc content at the beginning of the chase C_0_, and zinc content after 40 min C_40_ were determined, ZP1 and ZP2 in parallel experiments with isotope-enriched zinc (Tables 1 to 4)

**TABLE 1:** Pulse-chase with *C. metallidurans* strain AE104^a^

Chase		None	Zn(II)	Zn(II)	EDTA	Ni(II)	Co(II)	Cd(II)	Mn(II)	Mg(II)
Concentration		0	1 mM	100 µM						
mZn-TMM										
C_max_-uptake	1,000 /cell	263 ± 61	229 ± 48	250 ± 98	260 ± 36	204 ± 5	152 ± 7	160 ± 10	215 ± 14	174 ± 18
v_up_(0)	1 /s	145 ± 34	153 ± 32	115 ± 45	150 ± 21	165 ± 4	195 ± 9	187 ± 11	161 ± 11	184 ± 19
Coefficient		99.6%	99.5%	99.2%	99.8%	100.0%	99.9%	99.9%	99.9%	99.7%
C_o_-efflux	1,000 /cell	*86 ± 1*	146 ± 2	170 ± 2	255 ± 4	156 ± 1	165 ± 1	*90 ± 0*	141 ± 0	120 ± 1
v_eff_(0)	1 /s	−14 ± 0	64 ± 1	112 ± 1	214 ± 3	57 ± 0	79 ± 1	*−5 ± 0*	42 ± 0	20 ± 0
Coefficient		*79.7%*	93.9%	98.9%	98.2%	99.2%	98.5%	*−69.0%*	99.2%	93.7%
Low metal lZn_lMg										
C_max_-uptake	1,000 /cell	315 ± 27	289 ± 27	319 ± 43	418 ± 59	486 ± 60	421 ± 64	456 ± 42	525 ± 102	327 ± 34
v_up_(0)	1 /s	1,430 ± 123	1,812 ± 167	1,355 ± 184	943 ± 134	836 ± 103	954 ± 144	894 ± 82	764 ± 149	1,336 ± 138
Coefficient		96.3%	92.5%	92.5%	97.5%	98.9%	97.2%	99.2%	98.0%	95.8%
C_o_-efflux	1,000 /cell	290 ± 1	354 ± 4	495 ± 5	512 ± 5	529 ± 6	483 ± 6	323 ± 1	535 ± 4	372 ± 4
v_eff_(0)	1 /s	27 ± 0	141 ± 2	314 ± 3	285 ± 3	271 ± 3	251 ± 3	18 ± 0	273 ± 2	142 ± 2
Coefficient		88.2%	94.6%	98.4%	97.5%	96.6%	96.1%	80.2%	98.2%	93.5%

^a^ *C. metallidurans* strain AE104 was cultivated in moderate zinc (mZn) or low zinc-low magnesium (lZn_lMg) medium to a turbidity of 150 Klett units, harvested by centrifugation and resuspended in uptake buffer. The cells were kept in ice until needed. Na gluconate was added to a final concentration of 2 g/L, the experiment started by adding 1 µM ^65^Zn (60 nCi/mL), and the cells were incubated with shaking at 30°C. Samples (500 µL) were removed after 0.25, 5, 10, and 15 min. At 20 min, non-radioactive Zn(II), other metal cation, or EDTA was added and samples were removed at 20.25, 25, 30, 35, and 40 min. The samples were filtrated through membrane filters with a pore diameter of 0.2 µm and immediately washed twice with 5 mL ice-cold washing buffer (50 mM Tris-HCl, pH 7.0; 50 mM EDTA). Radioactivity was measured using a scintillation counter. The number of ^65^Zn atoms per cell was determined from the cpm values, the number of cells per mL, and the total radioactivity in 100 µL cell suspension. At least three biologically independent experiments were done. The number of ^65^Zn per cell before the chase was adapted in a Lineweaver-Burke-like plot to the function “1 /C = 1/C_max_ + t_0.5_/C_max*_t.” v_up_ is the first deviation of time at t = 0 of this function, v_u_p = C_max_/t_0.5_. After the chase, the number of ^65^Zn per cell was adapted to an exponential decay-curve “C = C_o *_ e^(τ_*_t)” with t_0.5_ =ln(2)/τ. v_eff_ is the first deviation of time at t = 0 of this function, v_eff_ = −τ_*_C_0_. The regression coefficients of these curve fittings are given. Numbers in italics indicate a coefficient <80%.

**TABLE 2:** Summary of the ^65^Zn pulse-chase experiments^a^

Medium	Uptake (pulse)			Chase		Pulse continued (control)	
Strains	v_up_ (s^−1^) , % AE104	C_20_, 1000 Zn/cell	C_max_/C_20_	v_eff_ (s^−1^), % AE104	C_40_/C_20_	v_eff_ (s^−1^), % AE104	C_40_/C_20_
Moderate Zn							
AE104	162 ± 25; 100% ± 15%	96 ± 7; 100% ± 7%	2.20	112.3 ± 1.0; 100.0% ± 0.9%	37.2% ± 6.7%	−14.4 ± −0.2; −12.8% ± −0.2%	127.0% ± 8.8%
Chase with 1 mM				63.9 ± 0.9; 58.9% ± 0.8%	57.4% ± 5.7%		
AE104, ^63^Ni	934 ± 551	93 ± 12 (10^3^ Ni/cell)	1.05	8.7 ± 0.0; 100.0% ± 0.1%	86.0% ± 6.3%	−13.2 ± 0.0; −152.0% ± −0.2%	107.6% ± 19.8%
∆*zupT*	94 ± 7; 58% ± 4%	64 ± 4; 66% ± 4%	2.67	38.8 ± 0.3; 34.5% ± 0.2%	46.4% ± 12.7%	2.1 ± 0.0; 1.9% ± 0.0%	102.5% ± 2.5%
∆7	211 ± 117; 131% ± 73%	37 ± 6; 39% ± 6%	1.37	11.4 ± 0.1; 10.2% ± 0.1%	71.8% ± 11.2%	5.8 ± 0.0; 5.2% ± 0.0%	67.6% ± 8.7%
∆9	356 ± 57; 220% ± 36%	88 ± 2; 91% ± 2%	1.46	15.0 ± 0.1; 13.3% ± 0.1%	77.5% ± 2.2%	12.3 ± 0.0; 10.9% ± 0.0%	83.9% ± 25.4%
∆e2	84 ± 4; 52% ± 2%	73 ± 2; 76% ± 2%	5.54	29.0 ± 0.1; 25.8% ± 0.1%	64.5% ± 7.3%	−18.3 ± 0.0; −16.3% ± 0.0%	133.0% ± 6.4%
∆e4	68 ± 8; 42% ± 5%	62 ± 1; 64% ± 1%	8.34	18.5 ± 0.0; 16.5% ± 0.0%	70.2% ± 5.2%	−9.7 ± 0.0; −8.7% ± 0.0%	117.5% ± 6.9%
∆*ppk*	71 ± 30; 44% ± 19%	45 ± 8; 47% ± 8%	4.72	13.2 ± 0.0; 11.7% ± 0.0%	85.6% ± 29.6%	−22.5 ± −0.2; −20.0% ± −0.2%	159.6% ± 52.3%
∆*gshA*	63 ± 10; 39% ± 6%	42 ± 0; 44% ± 0%	2.67	26.9 ± 0.3; 23.9% ± 0.3%	37.9% ± 8.5%	−5.1 ± 0.0; −4.5% ± 0.0%	111.6% ± 60.5%
CH34	146 ± 17; 90% ± 11%	30 ± 1; 31% ± 1%	1.05	4.7 ± 0.0; 4.2% ± 0.0%	83.4% ± 29.7%	−10.3 ± 0.0; −9.2% ± 0.0%	148.9% ± 49.9%
Chase with 1 mM				11.0 ± 0.1; 9.8% ± 0.0%	63.6% ± 6.5%		
CH34 induced	69 ± 11; 43% ± 7%	27 ± 5; 28% ± 6%	1.75	0.5 ± 0.0; 0.5% ± 0.0%	122.3% ± 62.6%	−12.8 ± −0.1; −11.4% ± −0.1%	169.0% ± 74.9%
Chase with 1 mM				16.0 ± 0.2; 14.2% ± 0.2%	53.2% ± 35.5%		
Low zinc							
AE104	227 ± 17; 100% ± 8%	92 ± 3; 100% ± 3%	1.62	125.4 ± 1.3; 100.0% ± 1.0%	45.2% ± 16.6%	−11.2 ± −0.1; −8.9% ± −0.1%	128.1% ± 10.4%
∆*zupT*	67 ± 1; 30% ± 0%	63 ± 4; 68% ± 4%	7.44	15.4 ± 0.1; 12.2% ± 0.0%	67.7% ± 2.1%	−6.7 ± 0.0; −5.3% ± 0.0%	118.8% ± 12.9%
Low Zn and Mg							
AE104	1147 ± 351; 100% ± 31%	272 ± 28; 100% ± 10%	1.45	314.4 ± 3.0; 100.0% ± 0.9%	40.2% ± 8.6%	27.1 ± 0.1; 8.6% ± 0.0%	83.8% ± 10.7%
AE104, Ni	1,911 ± 218	387 ± 22	1.26	63.5 ± 0.1; 100.0% ± 0.1%	77.6% ± 6.2%	−12.7 ± 0.0; −19.9% ± 0.0%	100.6% ± 12.5%
∆*zupT*	509 ± 10; 44% ± 1%	115 ± 4; 42% ± 1%	1.38	53.3 ± 0.2; 16.9% ± 0.1%	52.5% ± 12.3%	−2.1 ± 0.0; −0.7% ± 0.0%	101.4% ± 11.4%
∆7	212 ± 39; 18% ± 3%	45 ± 6; 17% ± 2%	1.46	9.5 ± 0.0; 3.0% ± 0.0%	65.8% ± 12.7%	4.6 ± 0.0; 1.5% ± 0.0%	96.5% ± 5.9%
∆9	281 ± 189; 24% ± 16%	50 ± 12; 18% ± 4%	1.40	6.6 ± 0.0; 2.1% ± 0.0%	69.0% ± 2.3%	2.9 ± 0.0; 0.9% ± 0.0%	100.9% ± 16.6%
∆e2	812 ± 66; 71% ± 6%	244 ± 4; 90% ± 2%	1.42	85.0 ± 0.1; 27.0% ± 0.0%	67.3% ± 10.0%	34.6 ± 0.1; 11.0% ± 0.0%	88.6% ± 5.5%
∆e4	479 ± 1; 42% ± 0%	197 ± 27; 73% ± 10%	1.59	28.5 ± 0.0; 9.1% ± 0.0%	74.3% ± 4.3%	14.9 ± 0.0; 4.7% ± 0.0%	104.7% ± 7.9%
∆*ppk*	378 ± 60; 33% ± 5%	177 ± 8; 65% ± 3%	2.04	81.3 ± 0.3; 25.9% ± 0.1%	55.1% ± 17.5%	−6.3 ± 0.0; −2.0% ± 0.0%	110.8% ± 6.7%
∆*gshA*	797 ± 407; 69% ± 36%	99 ± 15; 36% ± 6%	1.23	50.6 ± 0.4; 16.1% ± 0.1%	40.6% ± 9.6%	−0.1 ± 0.0; 0.0% ± 0.0%	106.2% ± 13.9%

^*a*^ As in Table 1 but additionally low zinc medium lZn was used for strain AE104 and its ∆*zupT* mutant.

**TABLE 3:** Pulse with ^67^Zn, chase with other metals in strain AE104^*a*^

Medium	10^3^ Zn			Metal of chase^*b*^	All except chase^*c*^	
Chase	ZP1	ZP2	ZP1 + 2			
mZn-TMM						
Initial cells	76.3 ± 4.7	< 0 ± .2	76.2 ± 4.9			
^67^Zn-pulse	59.5 ± 0.7	22.4 ± 1.4	81.9 ± 2.1			
Chase Ni	54.9 ± 1.4	28.2 ± 0.4	83.2 ± 1.8	86.2 ± 2.9	3.3 ± 0.1	10^3^ Ni
Chase Co	50.4 ± 1.8	17.5 ± 1.1	67.8 ± 2.9	197 ± 8	9 ± 1	10^3^ Co
Chase Cd	47.8 ± 1.3	13.4 ± 0.3	61.2 ± 1.6	103 ± 3	0 ± 0	10^3^ Cd
Chase Mn	54.7 ± 0.7	33.2 ± 0.9	87.8 ± 1.6	44.1 ± 4.3	0.2 ± 0.1	10^3^ Mn
Chase Mg	52.4 ± 1.2	30.9 ± 1.0	83.3 ± 2.3	9.62 ± 0.17	9.64 ± 0.31	10^6^ Mg
Chase EDTA	50.1 ± 3.9	13.9 ± 1.1	64.0 ± 5.0			
lZn_lMg-TMM						
Initial cells	3.5 ± 1.1	0.0 ±< 0	3.5 ± 1.1			
^67^Zn-pulse	4.7 ± 0.3	70.6 ± 5.5	75.3 ± 5.8			
Chase Ni	7.3 ± 0.8	46.8 ± 1.0	54.2 ± 1.8	278 ± 6.3	5.0 ± 0.3	10^3^ Ni
Chase Co	5.7 ± 2.4	35.2 ± 1.8	40.9 ± 4.2	564 ± 15	0.3 ± 0.3	10^3^ Co
Chase Cd	5.1 ± 1.3	44.1 ± 0.3	49.2 ± 1.5	89.1 ± 7.0	0.3 ± 0.4	10^3^ Cd
Chase Mn	5.9 ± 1.1	71.2 ± 2.7	77.1 ± 3.8	43.6 ± 2.3	0.4 ± 0.3	10^3^ Mn
Chase Mg	5.4 ± 0.5	72.3 ± 1.9	77.8 ± 2.4	10.1 ± 0.4	10.1 ± 0.5	10^6^ Mg
Chase EDTA	4.5 ± 0.7	48.3 ± 2.8	52.8 ± 3.5			

^*a*^ The cells were cultivated in moderate zinc (mZn) or low zinc, low magnesium medium (lZn-lMg) and loaded with 1 µM ^67^Zn for 20 min at 30°C with shaking, followed by 100 µM of other metal cations or EDTA. Samples for the metal determination by ICP_MS were taken before and after the uptake period as well as after the chase.

^*b*^ This lists only the content of a metal cation used for the chase after the chase period with this metal.

^*c*^ This lists the mean values for all the other experiments. At least three experiments and deviation are indicated. Please note that the magnesium content is millions of atoms per cell, that of the other metal thousand atoms per cell.

**TABLE 4:** Summary of the experiments with stable ^67^Zn that accompanied the pulse-chase experiments with radioactive ^65^Zn^*a*^

Strains	Initial 10^3^ Zn			10^3^ Zn after pulse			10^3^ Zn after chase (0.1 mM)		
Medium	ZP1	ZP2; %ZPt	ZP1 +ZP2	ZP1	ZP2; %ZPt	ZP1 +ZP2	ZP1	ZP2; %ZPt	ZP1 +ZP2
Moderate Zn									
AE104	103 ± 9	0.0 ± 0.2; 0.0%	103 ± 9	76.6 ± 1.8	27.4 ± 2.2; 26.4%	104 ± 4	240 ± 20	6.1 ± 0.8; 2.5%	246 ± 21
Chase 1 mM							2,401 ± 420	6.3 ± 0.1; 0.3%	2,407 ± 420
∆*zupT*	42.8 ± 1.8	<0	42.6 ± 1.7	37.8 ± 2.1	20.5 ± 1.2; 35%	58.3 ± 3.3	226 ± 15	4.1 ± −0.1; 2%	230 ± 14
∆7	44.0 ± 2.3	<0	43.9 ± 2.2	36.5 ± 2.2	17.3 ± 0.6; 32%	53.8 ± 2.8	272 ± 22	4.5 ± 0.3; 2%	276 ± 22
∆9	40.3 ± 2.3	<0	40.2 ± 2.4	32.8 ± 2.0	13.2 ± 0.9; 29%	46.0 ± 2.9	204 ± 8	4.1 ± 0.0; 2%	209 ± 8
∆e2	91.5 ± 3.8	<0	91.2 ± 3.8	76.2 ± 3.9	27.5 ± 3.2; 27%	104 ± 7	535 ± 49	7.4 ± −0.9; 1%	542 ± 48
∆e4	84.4 ± 3.1	<0	84.2 ± 3.1	78.5 ± 4.8	23.2 ± 0.3; 23%	102 ± 5	502 ± 49	7.2 ± −0.3; 1%	509 ± 49
∆*ppk*	88.7 ± 4.1	<0	88.3 ± 4.1	67.4 ± 3.2	22.9 ± 3.3; 25%	90.4 ± 6.4	201 ± 19	4.1 ± 0.3; 2%	206 ± 19
∆*gshA*	75.2 ± 1.6	<0	75.0 ± 1.6	60.8 ± 1.1	15.4 ± 0.2; 20%	76.2 ± 1.3	179 ± 6	3.5 ± 0.2; 2%	183 ± 6
CH34	77.0 ± 1.8	<0	76.6 ± 1.8	65.9 ± 0.7	11.5 ± 0.7; 15%	77.4 ± 1.4	150 ± 3	3.2 ± 0.0; 2%	153 ± 3
Chase 1 mM							864 ± 32	<0	863 ± 32
CH34 induced	235 ± 11	<0	234 ± 10	192 ± 6	0.20 ± 0.04; 0%	192 ± 6	215 ± 4	<0	214 ± 4
Chase 1 mM							429 ± 31	<0	428 ± 31
Low Zn									
AE104	11.0 ± 1.9	0.0 ± 0.0; 0.1%	11.1 ± 1.9	6.7 ± 0.7	79.4 ± 5.3; 92.3%	86.1 ± 6.0	181 ± 17	24.6 ± 1.4; 12.0%	206 ± 19
Chase 1 mM							6,171 ± 118	24.1 ± −0.1; 0.4%	6,195 ± 118
∆*zupT*	43.2 ± 1.4	<0	43.0 ± 1.4	37.0 ± 0.8	30.8 ± 2.5; 45%	67.8 ± 3.3	190 ± 8	6.9 ± 0.2; 4%	197 ± 8
Low Zn and Mg									
AE104	29.5 ± 10.2	0.5 ± 0.0; 1.6%	30.0 ± 10.1	6.3 ± 1.3	85.0 ± 3.9; 93.1%	91.3 ± 5.3	201 ± 33	25.4 ± 2.0; 11.2%	226 ± 35
Chase 1 mM							5,261 ± 552	27.0 ± −1.1; 0.5%	5,288 ± 551
∆*zupT*	8.3 ± 0.8	<0	8.3 ± 0.8	8.0 ± 0.3	57.4 ± 3.0; 88%	65.4 ± 3.3	169 ± 22	13.9 ± 0.3; 8%	183 ± 22
∆7	10.4 ± 0.8	<0	10.3 ± 0.8	9.00 ± 1.4	67.4 ± 4.4; 88%	76.4 ± 5.8	248 ± 24	20.2 ± 1.1; 8%	268 ± 25
∆9	8.5 ± 1.4	0.0 ± 0.0; 0%	8.5 ± 1.4	8.00 ± 1.2	75.8 ± 10.8; 90%	83.8 ± 12.0	404 ± 67	19.2 ± 1.6; 5%	423 ± 68
∆e2	8.6 ± 0.6	0.0 ± 0.0; 0%	8.6 ± 0.6	8.8 ± 0.8	97.1 ± 3.7; 92%	106 ± 5	560 ± 18	38.2 ± 3.0; 6%	599 ± 21
∆e4	7.6 ± 0.8	0.0 ± 0.0; 0%	7.6 ± 0.8	8.1 ± 0.3	91.6 ± 5.1; 92%	99.7 ± 5.5	767 ± 100	37.1 ± −0.4; 5%	805 ± 99
∆*ppk*	7.7 ± 1.0	0.0 ± 0.0; 0%	7.7 ± 1.0	8.9 ± 0.6	75.7 ± 3.7; 89%	84.6 ± 4.3	161 ± 11	20.2 ± −0.1; 11%	181 ± 11
∆*gshA*	7.6 ± 0.2	<0	7.6 ± 0.2	10.4 ± 3.0	50.2 ± 2.9; 83%	60.5 ± 5.8	165 ± 21	16.5 ± 0.1; 9%	182 ± 21

^*a*^ The cells of the indicated *C. metallidurans* mutants were incubated in Tris-buffered mineral salts medium adjusted to 200 nM Zn(II) (moderate zinc), the same medium without trace element solution SL6 and 0.1 mM Mg(II) instead of 1 mM Mg(II) (low Zinc and Mg) or TMM medium without SL6 but 1 mM Mg(II) (low Zn). Zinc coming from SL6 or contaminations was in the natural isotope composition. These cells were incubated with 1 µM enriched stable ^67^Zn for 20 min (pulse) and subsequently chased with 100 µM Zn(II) (or 1 mM when indicated) with the natural isotope composition again. The zinc pools ZP1 and ZP2 were calculated from the ICP-MS measurements and ZPt = ZP1+ZP2 was determined.

The mean initial uptake velocity v_up_(0) of all these experiments was 162 ± 25 ^65^Zn cell^−1^ s^−1^ in mZn-TMM and 1,147 ± 351 ^65^Zn cell^−1^ s^−1^ under zinc and magnesium starvation conditions (Table 1), respectively, which was a sevenfold increase as previously published (21). Moreover, since the expected v_up_(0) at 1 µM Zn was 166 ± 94 Zn(II) s^−1^ cell^−1^, the measured v_up_(0) was in agreement with previously published experiments (21). Metal-starved cells displayed a higher initial zinc import rate than cells that had been cultivated under zinc-replete conditions.

Zinc-replete AE104 accumulated C_20_ = 96,000 ^65^Zn atoms within 20 min (Table 2). The ratio C_max_/C_20_ indicated that an equilibrium had not been reached within this time period. Consequently, the non-chased cells continued to accumulate ^65^Zn to a level of about 130,000 ^65^Zn per cell (Fig. 2). When chased with 100 µM non-radioactive zinc, the cellular ^65^Zn content decreased with an initial net efflux rate of 112 ± 1 ^65^Zn cell^−1^ s^−1^ to a level of 37% of the C_20_ level (C_40_/C_20_) at the end of the experiment. As measured with stable, isotope-enriched ^67^Zn, zinc-replete AE104 contained 103,000 zinc atoms per cell at the beginning of the uptake period (Table 4). After the uptake period, the total number of zinc ions remained the same; however, the initial zinc in ZP1 decreased to 76,600 Zn per cell and 26.4% of the zinc was now present in ZP2. After the chase with non-isotope-enriched zinc, the zinc content of the cells increased to 260,000 Zn per cell but only 2.5% resided in ZP2.

In zinc-replete AE104 cells, about 25% of the zinc ions were exchanged against incoming zinc ions at the uptake period. These zinc ions in ZP2 were subsequently exported again during the chase period. These data were in full agreement with a flow equilibrium governing the cellular zinc content. Zinc ions were constantly imported into and exported from the cells (Fig. 2).

Metal-starved AE104 cells that accumulated zinc with a sevenfold higher v_up_(0) compared to zinc-replete cells already reached an equilibrium of the cellular zinc content after 15 min at 272,000 ^65^Zn per cell (Fig. 2B; Table 2). After 20 min, even the non-chased control cells exported zinc with v_eff_(0) = 27 ^65^Zn cell^−1^ s^−1^, the cells chased with 100 µM non-radioactive zinc with 314 ± 3 ^65^Zn cell^−1^ s^−1^ (Tables 1 and 2). As determined with ^67^Zn, the initial zinc content at the onset of the experiment was about 30,000 Zn per cell, increased to about 90,000 Zn per cell after the uptake period with 1 µM ^67^Zn with most of this zinc residing in ZP2. Comparable to zinc-replete cells, about ¾ of the zinc in ZP2 was removed again in the subsequent chase period (Table 4).

To analyze, whether this increased uptake rate was caused by zinc or the magnesium starvation conditions in metal-starved cells, zinc-replete and -starved cells (mZn- and lZn-medium, respectively) were compared. There was no difference between these cells in pulse-chase experiments (Fig. S3) and the cells reached similar C_20_ values, although the initial zinc uptake rate was increased by 40% (D = 1.55) from 162 ± 25 to 227 ± 17 ^65^Zn cell^−1^ s^−1^ (Table 2). The initial zinc content of zinc-starved cells was only 11,000 Zn per cell, much lower than that of zinc-replete AE104 cells. Nevertheless, the influence of the decreased zinc content of the medium on the import and export rate of zinc in the pulse-chase experiment was much smaller than that of the lower magnesium content, although the lower magnesium content in the respective medium did not result in a lower magnesium content in the cells (Table S4).

Compared to zinc-replete cells and magnesium-replete but zinc-starved cells, zinc-magnesium-starved AE104 cells imported zinc with a sevenfold higher initial rate, reached equilibrium after 15 min at a threefold higher ^65^Zn level, exported zinc with a threefold higher rate in the subsequent chase period, thereby removing 60% (^65^Zn) or 70% (ZP2) of the previously imported zinc again (Tables 2 and 4). This was again evidence in favor of a flow equilibrium being in charge of the cellular zinc homeostasis and that the magnesium content of the medium was crucial for the up-regulation of the import rates.

### Other metals and EDTA

Metal ions that interfere with zinc uptake or complex the metal ion in the growth medium should influence the flow equilibrium because they should decrease the influx rates of zinc of residual ^65^Zn during the chase period. In addition to zinc, strain AE104 was also chased with 100 µM of other metal cations or EDTA after being loaded with 1 µM ^65^Zn or ^67^Zn (Tables 1 and 3; Fig. S2). In zinc-replete cells, the ^65^Zn content decreased when cells were chased with Mg(II) <Mn(II) <Ni(II) <Co(II) <EDTA (Fig. S2A), which was paralleled by an increase in the v_eff_(0) from Mg(II)- to EDTA-chased cells (Table 1). The efflux rate caused by a chase with 100 µM EDTA was 214 ^65^Zn cell^−1^ s^−1^ and twofold higher than the 112 ^65^Zn cell^−1^ s^−1^ after a chase with 100 µM non-radioactive zinc in zinc-replete cells. This indicated that the complexation of the remaining ^65^Zn in the medium by EDTA may have decreased the residual net import of ^65^Zn. This was no longer the case in zinc-starved cells. Cells chased with 100 µM Cd(II) did not decrease their zinc content during the chase period but the increase in this value was smaller than that of the control cells (Fig. S2A; Table 1).

Zinc-replete cells contained about 80,000 Zn ions in ZP1 at the onset of the experiment and remained at this total zinc level after the uptake period (Table 3, ^67^Zn-pulse), albeit with about 22,000 Zn ions now residing in ZP2 so that 25% of the zinc in ZP1 had been exchanged against incoming zinc. The chase resulted in a strong accumulation of the metals used for the chase, namely nickel, cobalt, cadmium, and manganese, but the magnesium content did not change (Table 3). The chase was also accompanied by a decreased total zinc content in ZP1 + ZP2 in the case of cobalt, cadmium, and EDTA but not after a chase with Mn, Mg, or Ni. The zinc level in ZP2 was decreased by EDTA and Cd, to a smaller level by Co, but increased when the cells were chased with Ni, Mg, or Mn (Table 3). All substances also decreased the zinc level in ZP1 between −20% (Cd) and −8% (Ni). In comparison, a chase with 100 µM Zn-depleted ZP2 down to 6,000 Zn per cell (Table 4). Compounds that interfered with zinc uptake most strongly (EDTA >Co) decreased the zinc content in ZP2 and those that interfered to a smaller degree (Ni, Mn, and Mg) increased this level, while all compounds decreased the zinc content in ZP1. There was a constant turnover of zinc in zinc-replete cells. Even when chased with other metal cations, zinc was still imported into the cells but uptake was affected by complexation (EDTA) or competition for uptake systems (Co).

In metal-starved cells of strain AE104, the other metal cations, and EDTA also interfered with ^65^Zn uptake (Tables 1 and 3; Fig. S2B). The net efflux rates caused by chase with Ni, Co, Mn, Mg, or EDTA were not much different from that of Zn, whereas cells chased by 100 µM Cd(II) were again comparable to the negative control, un-chased cells. The initial zinc content of these cells was below 7,300 Zn per cell in ZP1 (Table 3) and increased during the uptake phase to 71,000 Zn per cell, predominantly residing in ZP2. While a chase with other metal cations or EDTA did not change the low zinc content in ZP1, that in ZP2 decreased after a chase with Co > Cd > Ni >EDTA but not with Mn or Mg. Except for Mg, again, the chasing metals were accumulated by the cells, Co even to a very high level of 564,000 ± 15,000 Co per cell (Table 3).

Zinc-replete and metal-starved AE104 cells were also incubated in the presence of 1 µM ^63^Ni and chased with 100 µM non-radioactive nickel. The cellular nickel content decreased compared to the non-chased control (Fig. S4; Table S5). A chase with 100 µM non-radioactive zinc also decreased the cellular nickel level but to a smaller degree than a chase with nickel. This demonstrated the existence of a kinetical flow equilibrium also for nickel ions. An accompanying experiment using the ICP-MS determination of the cellular metal content verified that strain AE104 was able to accumulate Mn, Co, Ni, and Cd under both physiological conditions (Table S5); however, accumulation of Mn and Ni in zinc-replete cells was only to a small extend.

The data coming from chase experiments with other metal cations or substances, which interfered with zinc uptake, again presented evidence for the flow equilibrium. A net efflux of zinc could also be observed under these conditions (Fig. S2). Ni, Mn, and Mg did not remove zinc from ZP1 or ZP2 (Table 3). This was also true for Mn and Mg under metal-starvation conditions but no longer for Ni, which accumulated to levels of 278,000 Ni per cell and removed 1/3 of the zinc from ZP2. A chase with cobalt ions decreased the zinc content in ZP1 and ZP2 in zinc-replete cells and ZP2 in metal-starved cells (Table 3) but much less than zinc itself (Table 4). Cd was a special case, with only minor interference with zinc uptake but a strong depletion of zinc from the zinc pools. This was probably based on the up-regulation of the cadmium-exporter CadA by CadR since CadA and ZntA are both exporting zinc from *C. metallidurans* cells (7, 19). In total, Co and to a lesser degree Ni seemed to interfere not only with zinc uptake but may also mobilize Zn from its binding sites for subsequent export.

### Role of uptake systems

Removal of zinc uptake systems should affect the cellular zinc turnover. ZupT of the ZIP protein family is the only zinc-regulated uptake system in *C. metallidurans* and is under the control of the zinc uptake regulator Zur [Fig. 1, (7–9, 25, 26, 33)]. Pulse-chase experiments were performed with zinc-replete, zinc-starved, and metal-starved cells of the ∆*zupT* strain of *C. metallidurans* AE104. Zinc-starved and -replete ∆*zupT* cells imported ^65^Zn with half of the uptake rate and 2/3 of the C_20_ value of the parent cells. As with the parent, the difference between the two differently cultivated ∆*zupT* cells was small. Although the pulse-chase curves are not much different from each other (Fig. S3) but resulted in slightly different parameters (Table 2). While the v_up_(0) increased from zinc-replete to -starved parent cells, the v_up_(0) in ∆*zupT* cells decreased from 94 ^65^Zn cell^−1^ s^−1^ to 67 ^65^Zn cell^−1^ s^−1^. The subsequent efflux rates in the chase period were similar to parent cells but decreased in ∆*zupT* cells from 38.8 ^65^Zn cell^−1^ (moderate Zn) to 15.4 ^65^Zn cell^−1^ (low Zn, Table 2). As has also been observed with the parent cells, the influence of zinc starvation on the flow equilibrium was much smaller than that of magnesium-zinc starvation. Consequently, only cells cultivated in moderate zinc medium mZn and low zinc-low magnesium medium lZn_lMg were subsequently compared with each other.

In the accompanying ^67^Zn experiments (Table 4), zinc-replete *∆zupT* cells contained half of the zinc content of the parent at the beginning of the experiment, lost only a smaller number of zinc atoms from ZP1 during the uptake phase but gained 20,000 ^65^Zn cell^−1^ in this period, resulting in a net increase in the cellular zinc content. Nevertheless, zinc-replete ∆*zupT* cells contained only half as much zinc compared to the parent after the uptake phase (Table 4). These experiments revealed an important role of ZupT in zinc homeostasis, although ZupT was not essential for zinc import into *C. metallidurans*. It was responsible for 42% of the uptake rate in zinc-replete and 70% in zinc-starved cells. As indicated by the C_max_/C_20_ values, none of these cells reached equilibrium after the uptake period. Especially zinc-starved ∆*zupT* cells were far from equilibrium (C_max_/C_20_ = 7.44, Table 2). Consequently and due to the lower import rate, the ∆*zupT* cells reached only 2/3 of the ^65^Zn content of the parent cells and exported at a lower rate during the chase period. Despite a similar C_20_- and also a similar ZP1 + ZP2- level of zinc-replete and -starved ∆*zupT* cells, the efflux rate of the starved cells was only 40% of that of the replete cells. This indicated that regulatory processes of the cellular zinc homeostasis might originate from ZupT or ZupT-dependent zinc import.

**Fig 3:**
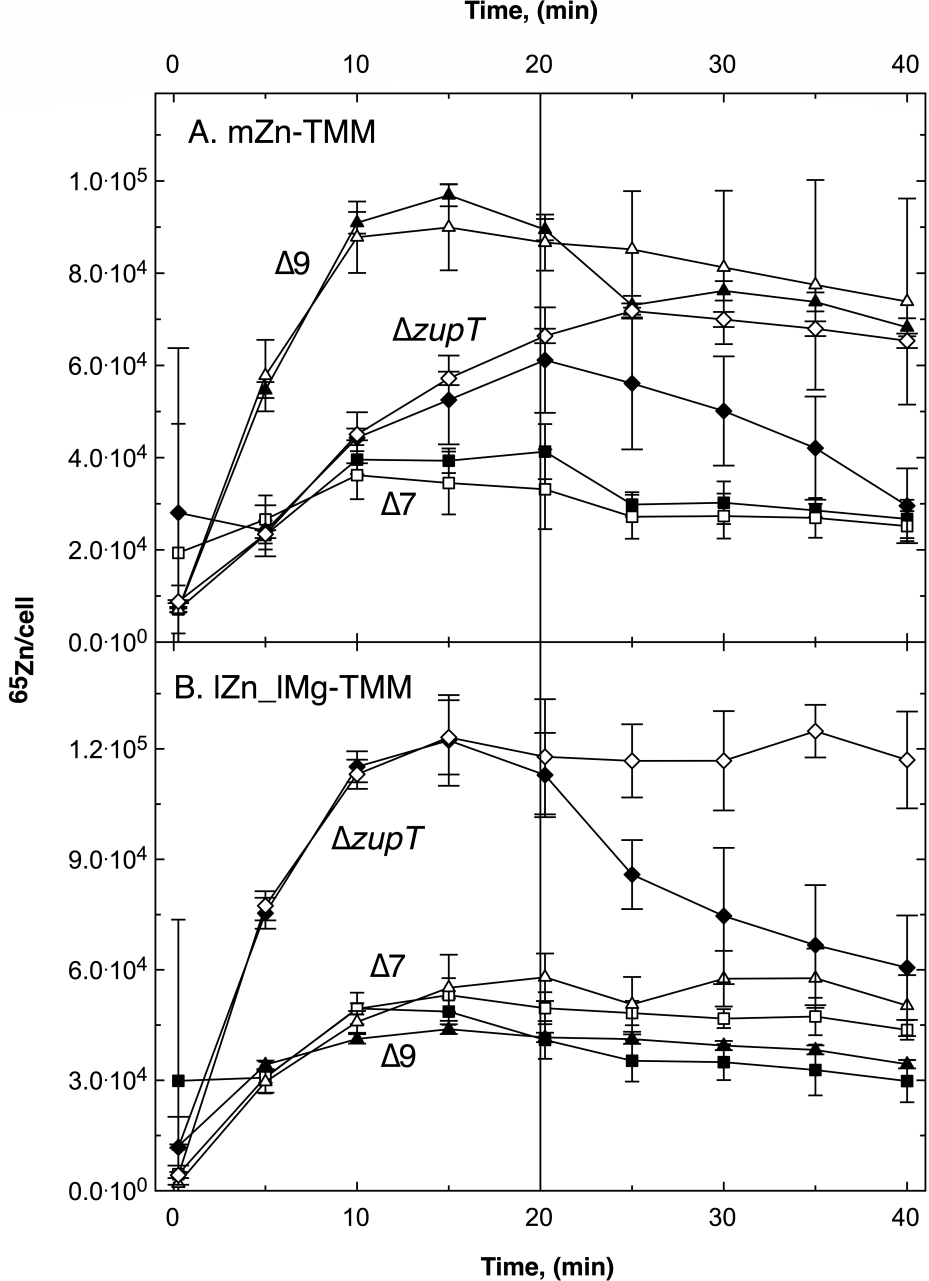
Pulse-chase experiment with *C. metallidurans* AE104 uptake mutants ∆*zupT*, ∆7 and ∆9. Cells of strain ∆*zupT* (diamonds), ∆7 (*∆zupT ∆corA1 ∆corA2 ∆corA3 ∆zntB ∆pitA ∆hoxN,* squares), and ∆9 (∆7 *∆mgtA ∆mgtB::kan,* triangles) were cultivated in TMM containing 200 nM zn(II) (mZn-TMM, Panel A) or no added Zn(II) and 0.1 mM Mg(II) (lZn_lMg, Panel B) as described in Fig. 2. Pulse at t = 0 with 1 µM ^65^Zn(II), chase at t = 20 min with 100 µM non-radioactive Zn(II) (black symbols), or not chased (open symbols). Figure S5 provides an additional comparison with and without parent strain AE104.

Zn-Mg-starved ∆*zupT* cells also accumulated ^65^Zn to a smaller extent than the parent cells (Fig. S5; Fig. 3; Table 2). ZupT was responsible for 56% of the initial import rate. As indicated by the pulse-chase curves and the C_max_/C_20_ values, metal-starved ∆*zupT* and parent cells were closer to an import-export equilibrium than cells cultivated under replete conditions.

When the genes for further uptake systems were deleted leading to the ∆7 (*∆zupT ∆corA1 ∆corA2 ∆corA3 ∆zntB ∆pitA ∆hoxN*) and ∆9 (∆7 *∆mgtA ∆mgtB::kan*) mutants, ^65^Zn import into metal-starved (lZn_lMg) cells decreased even more compared to the ∆*zupT* mutant (Fig. 3B; Table 2) and was on a similar level. The initial import rate decreased from 509 ^65^Zn cell^−1^ s^−1^ in the ∆*zupT* mutant to about 250 ^65^Zn cell^−1^ s^−1^ in ∆7 and ∆9, reaching an equilibrium after 20 min of uptake of C_20_ = 45,000 to 50,000 ^65^Zn cell^−1^ in ∆7 and ∆9 cells. The chased and control cells subsequently exported ^65^Zn again with net efflux rates of 9.5 ^65^Zn cell^−1^ s^−1^ (chased ∆7), 6.6 ^65^Zn cell^−1^ s^−1^ (chased ∆9), 4.6 ^65^Zn cell^−1^ s^−1^ (∆7 control), and 2.9 ^65^Zn cell^−1^ s^−1^ (∆9 control). The number of ^65^Zn atoms per cell in both mutants was much lower than that of the ∆*zupT* cells (C_20_ = 115,000 ± 4,000 ^65^ Zn cell^−1^), which corresponded to 65,400 ± 5,800 Zn cell^−1^ in ZP1 +ZP2 after 20 min (Table 4). In comparison, this ZP1 + ZP2-level after 20 min was higher in ∆7 and ∆9 cells, about 80,000 Zn cell^−1^. These zinc ions were mostly residing in ZP2 and had been imported during the uptake period (Table 4). Metal-starved ∆7 and ∆9 cells were still able to accumulate sufficient levels of zinc when the metal was provided at a concentration of 1 µM.

In zinc-replete ∆7 cells, zinc import was also lower than that in ∆*zupT* and parent cells. Despite a duplication of the v_up_(0) value from ∆*zupT* to ∆7, only half of the C_20_ value was reached (Table 2). The decrease in ZP1 and the total ^67^Zn content during the uptake phase was similar in both cells but the ZP2 level of the ∆7 cells was only 84% of that of the ∆*zupT* cells (Table 4). Since the initial efflux rate of 11.4 ^65^Zn cell^−1^ s^−1^ in ∆7 cells was much lower than that of ∆*zupT* cells, rapid down-regulation of zinc import during the uptake phase rather than up-regulation of efflux was responsible for the decreased zinc import in zinc-replete ∆7 cells.

Surprisingly, initial zinc import into zinc-replete ∆9 cells was much higher than that of parent, ∆*zupT* and ∆7 cells (Fig. 3; Fig. S5A, triangle compared to circles) with the highest measured ^65^Zn import rate of 356 ^65^Zn cell^−1^ s^−1^ in all zinc-replete cells (Table 2). The cells rapidly reached an equilibrium after 15 min. The subsequent initial efflux rate was not much enhanced compared to ∆7 cells (Table 2). After the uptake period, the ∆9 cells had not accumulated more ^67^Zn than before this time and contained only 60% of the ZP2 level of ∆*zupT* cells. This indicated an unexpected feature of the unknown zinc importer “X” (Fig. 1), which was activated under zinc-replete but not metal-starved conditions in ∆9 cells only and rapidly switched off when zinc became available.

These data demonstrated again that all nine systems and “X” (Fig. 1) were involved in zinc uptake in *C. metallidurans* (25, 30, 33, 34). “X” might be a third magnesium import system besides MgtA and MgtB with a broad substrate specificity.

### Role of efflux systems

**Fig 4:**
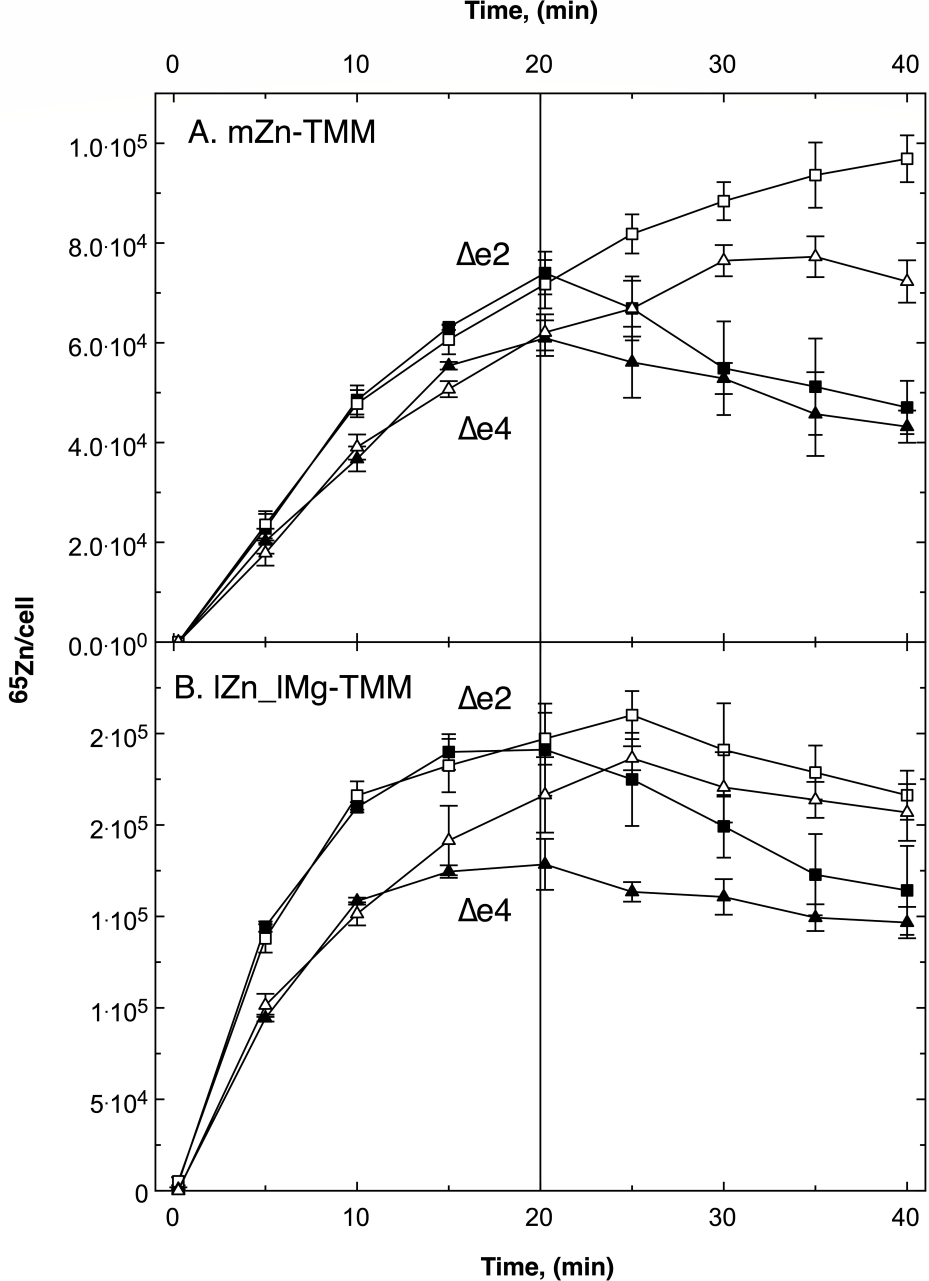
Pulse-chase experiment with *C. metallidurans* AE104 efflux mutants ∆e2 and ∆e4. Cells of strain ∆e2 (*∆zntA ∆cadA,* squares) and ∆e4 (∆e2 *∆dmeF ∆fieF,* triangles) were cultivated in TMM containing 200 nM Zn(II) (mZn-TMM, Panel A) or no added Zn(II) and 0.1 mM Mg(II) (lZn_lMg, Panel B) as described in Fig. 2. Pulse at t = 0 with 1 µM ^65^Zn(II), chase at t = 20 min with 100 µM non-radioactive Zn(II) (black symbols), or not chased (open symbols). Figure S6 provides an additional comparison with parent strain AE104.

The four known efflux systems ZntA, CadA, DmeF, and FieF (Fig. 1) may export zinc from cells of *C. metallidurans* AE104. ZntA and CadA are the main contributors because the zinc resistance of the double mutant ∆e2 (∆*zntA ∆cadA*) is 7.7 ± 0.6 µM zinc only slightly higher than that of the quadruple mutant ∆e4 (∆*zntA ∆cadA ∆dmeF ∆fieF*) with 7.1 ± 0.7 µM; the IC_50_ value of the parent is 1,056 ± 28 µM (19). Zinc-replete cells of the ∆e2 mutant imported ^65^Zn with half of the initial uptake rate of the parent and ∆e4 mutants with 42%, reaching C_20_ of 76% and 64% of the parent values, respectively (Table 2). Both mutants displayed a clear chase reaction (Fig. 4) with initial net efflux rates of 26% and 16% of the parent (Table 2; Fig. S6). The initial zinc content, accumulation of zinc in ZP2 during the uptake period, and removal of zinc from ZP2 during the chase period were not much different from the parent; however, the mutants accumulated large amounts of zinc during the chase period, more than 500,000 Zn per cell (Table 4). The absence of the two major or all known four zinc efflux systems increased zinc availability at the onset of the experiment, leading to decreased initial import rates, but even the ∆e4 mutant displayed a residual efflux reaction during the chase period.

In metal-starved cells, accumulation of zinc during the uptake phase was decreased in the efflux mutants compared to the parent (Fig. S6), in the ∆e4 mutant more than in the ∆e2 mutant (Fig. 4B). The ∆e2 mutant imported zinc with 71% of the import rate of the parent, reaching 90% of its C_20_ value, and exported the metal during the chase period with 27% of the net efflux rate (Table 2). Import into the ∆e4 mutant was much slower with 42% of the initial import rate of the parent but reached a C_20_ value of 73%. The ∆e4 mutant still exported zinc during the chase period with 9% of the net efflux rate of the parent. The initial zinc content of metal-starved mutant cells and the accumulated amount of zinc in ZP2 during the uptake period were comparable to the parent but more zinc was left in ZP2 after the chase period, which again resulted in a high amount of zinc bound by the mutant cells, nearly 600,000 in strain ∆e2 and 805,000 in strain ∆e4 (Table 4).

The four efflux systems ZntA, CadA, but unexpectedly also DmeF and FieF, clearly contributed to the efflux of zinc from cells of *C. metallidurans*, the P-type ATPases ZntA and CadA with 74% of the initial net efflux rate in zinc-replete cells and DmeF and FieF with about 10%. At least one additional efflux activity seems to exist in strain AE104. The contribution of efflux systems was in agreement with a flow equilibrium of zinc in *C. metallidurans*.

### Impact of metal-binding substance in the cytoplasm

**Fig 5:**
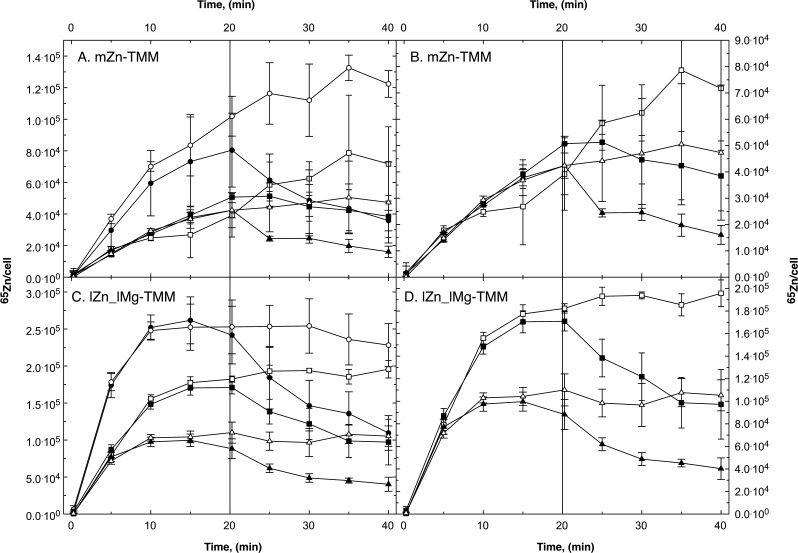
Pulse-chase experiment with *C. metallidurans* AE104 pool mutants ∆*ppk::kan* and ∆*gshA::kan*. Cells of strain AE104 (circles), ∆*ppk::kan* (squares), and ∆*gshA::kan* (triangles) were cultivated in TMM containing 200 nM zn(II) (mZn-TMM, Panels A and B) or no added Zn(II) and 0.1 mM Mg(II) (lZn_lMg, Panels C and D) as described in Fig. 2. Pulse at t = 0 with 1 µM ^65^Zn(II), chase at t = 20 min with 100 µM non-radioactive Zn(II) (black symbols), or not chased (open symbols). Panels B and D contain the same data as Panels A and C except the AE104 values.

Metal-binding sites in proteins including ribosomal ones, the cellular thiol glutathione GSH, and polyphosphate may sequester cytoplasmic zinc ions and interfere with its homeostasis (Fig. 1). In the case of GSH and polyphosphate, this influence was studied using the well-characterized ∆*gshA* (39) and ∆*ppk* (40) mutants of *C. metallidurans* strain AE104. In zinc-replete cells, the uptake phase of both mutants was not different from each other but different from the parent strain (Fig. 5). The initial uptake rates of both strains were similar and half of that of the parent strain AE104, leading to C_20_ values of 42,000 to 45,000 ^65^Zn per cell (Table 2). As indicated by the C_max_/C_20_ values, neither strain had reached an equilibrium after 20 min. Consequently, both strains continued a net import of ^65^Zn in the non-chased control (Table 2; Fig. 5). Both strains displayed net ^65^Zn efflux during the chase period. The ∆*gshA* strain with 27 ^65^Zn cell^−1^ s^−1^ and removing 62% of the ^65^Zn during the chase, the ∆*ppk* strain with 13 ^65^Zn cell^−1^ s^−1^ and removing 14%.

Zinc-replete ∆*gshA* and ∆*ppk* cells initially contained about 80,000 zinc atoms in ZP1 (Table 4). This content remained the same during the uptake period but with about 20,000 Zn per cell now in ZP2. During the chase period, zinc was depleted from ZP2 and the cells reached values after 40 min of about 200,000 zinc per cell, respectively. All these values were smaller compared to the respective AE104 data, the ∆*gshA* 56% and ∆*ppk* 84% of the value in parent cells.

In metal-starved cells, the ∆*ppk* mutant accumulated much more ^65^Zn than the ∆*gshA* cells (Fig. 5) but still on a lower level compared to the parent. The initial uptake rates of the ∆*ppk* mutant were 1/3 but they accumulated 2/3 of the C_20_ value of the parent AE104 (Table 2). The initial uptake rate of the *∆gshA* mutant was 69% but the C_20_ value was only 36% of that of the parent. The C_max_/C_20_ ratio for both strains indicated that an equilibrium had not been reached after 20 min. Consequently, both strains continued to import zinc in the negative control, while the parent exported ^65^Zn before the end of the uptake period. The net efflux velocities were 26% (∆*ppk*) and 16% (∆*gshA*) of that of the parent.

Metal-starved cells of both mutant strains possessed a zinc level below 8,000 Zn per cell at the onset of the experiment (Table 4), imported zinc during the uptake phase exclusively into ZP2, and removed about 2/3 of the zinc from ZP2 during the chase period. With respect to other metals (Table S4), ∆*gshA* cells contained a lower iron, cobalt, and nickel content in both media, and the ∆*ppk* cells had a higher nickel content in metal-starved cells.

Polyphosphate and glutathione were involved in zinc homeostasis. The lower initial uptake rates and C_20_ levels reached in the mutant cells compared to the parent suggested that zinc was more available for efflux during the turnover of the metal, which would be in agreement with a sequestration of zinc by both compounds in the cytoplasm. The metal-binding capacity of the cytoplasm interacted with the flow equilibrium to adjust the cytoplasmic zinc content.

### *C. metallidurans* CH34 wild type

Compared to strain AE104, the CH34 wild type contains a variety of plasmid-encoded metal transport systems (Fig. 1), which should affect the flow equilibrium of zinc. When the plasmid-free strain AE104 was chased with 1 mM non-radioactive zinc instead of 0.1 mM, efflux of ^65^Zn decreased at the higher zinc concentration used for the chase (Fig. 2, closed squares compared to circles), although the difference was very small and not significant in case of the metal-starved cells (Fig. 2B). The initial efflux velocity decreased from 112 ± 1 ^65^Zn cell^−1^ s^−1^ to 64 ^65^Zn cell^−1^ s^−1^ in zinc-replete and from 314 ^65^Zn cell^−1^ s^−1^ to 141 ^65^Zn cell^−1^ s^−1^ in metal-starved cells (Table 1). Because 1 mM should compete more efficiently than 100 µM non-radioactive zinc with uptake of the 1 µM ^65^Zn during the chase period and should mediate in a better mobilization of cytoplasmic ^65^Zn for efflux, this result was not expected. It may indicate an inhibition of the chased cells by 1 mM Zn(II). Indeed, the metal content of the AE104 cells after chase with 1 mM Zn(II) was 2.4 million (zinc-replete) or even 5.2 million (metal-starved) zinc per cell (Table 4).

*C. metallidurans* CH34 wild type should be capable of handling this problem. It contains in comparison to strain AE104 the plasmid-encoded efflux systems such as the CzcCBA transenvelope efflux system (Fig. 1). The CzcCBA system is also present under non-challenging conditions, growth in TMM without the addition of zinc (41). In pulse-chase experiments, zinc-replete CH34 wild type accumulated only 30,000 ^65^Zn during the uptake phase, and the C_max_/C_20_ ratio of 1.05 indicated that an equilibrium had been reached at 20 min (Table 2; Fig. S7). The v_up_(0) of CH34 was 146 ± 17 ^65^Zn cell^−1^ s^−1^ and not different from the AE104 values over all experiments of 162 ± 25 ^65^Zn cell^−1^ s^−1^ (Table 2). The CzcCBA system did not prevent zinc uptake into the cytoplasm at low zinc concentrations but strain CH34 was able to adjust rapidly to changing zinc availability. In contrast to strain AE104, the initial net efflux velocity more than doubled in strain CH34 from 4.7 ^65^Zn cell^−1^ s^−1^ to 11.0 ^65^Zn cell^−1^ s^−1^ when these cells were chased with 1 mM instead of 100 µM Zn(II) (Table 2). These values were only 9.8% and 4.2%, respectively, of the efflux velocity of AE104 cells chased with 100 µM zinc. Concerning the pulse-chase curves, these differences were not significant in non-induced CH34 cells (Fig. S7A) but the calculated v_eff_ values clearly were significant (Table 2).

When *C. metallidurans* was additionally cultivated in the presence of 0.1 mM Zn(II) used for an up-regulation of its efflux systems (Fig. S7; Tables 2 and 4; “CH34 induced”), again about 30,000 ^65^Zn were accumulated during the uptake period. There was no longer a significant ^65^Zn efflux when these cells were subsequently chased with 0.1 mM Zn(II) but when the cells were chased with 1 mM Zn(II), efflux was 16.0 ± 0.2 ^65^Zn cell^−1^ sec^−1^. CH34 cells chased with 1 mM Zn(II) possessed only 863,000 Zn(II) per cell compared to 2.4 million in strain AE104, and CH34 cells pre-cultivated in 0.1 mM Zn even less, 428,000 Zn(II) per cell (Table 4). These cells carried already 235,000 Zn per cell at the onset of the experiment in ZP1 and nearly no zinc in ZP2 (200 ± 40 zinc per cell) after the uptake period. After a chase with 0.1 mM Zn(II), they kept their zinc content at about 200,000 Zn per cell and only doubled the zinc content when chased with 1 mM zinc.

The plasmid-encoded Czc system is responsible for an enhanced efflux capability of *C. metallidurans* CH34 compared to the plasmid-free strain AE104 (42–44). The pulse-chase experiment (Fig. S7; Tables 2 and 4) clearly demonstrated the outstanding ability of strain CH34 to shield its cells against high zinc concentrations.

## DISCUSSION

### Zinc homeostasis in *C. metallidurans* is controlled by a flow equilibrium of uptake and efflux reactions

The plasmid-free *C. metallidurans* strain AE104 clearly demonstrated a constant turnover of cell-bound zinc. Uptake of ^65^Zn ions into zinc-replete cells had been investigated before (21) and occurred at 1 µM Zn(II) with an initial velocity of 166 ± 94 Zn(II) s^−1^ cell^−1^. This value was reproduced as 162 ± 25 ^65^Zn cell^−1^ s^−1^, and also a sevenfold increase in uptake velocity in metal-starved cells. Responsible for this was a lowered magnesium concentration rather than zinc starvation conditions. Magnesium is a competitive inhibitor of zinc uptake in *C. metallidurans* (21) so up-regulation of magnesium importers was responsible for the increased uptake rate in metal-starved cells.

When the cells, which accumulated ^65^Zn during an uptake period, were chased with 100 µM non-radioactive zinc, the net efflux of ^65^Zn was visible (Fig. 2). Adding 100 µM Zn(II) increased the zinc concentration to 101 µM. With a *K*_m_ of 137 ± 87 µM of the zinc import, this results according to the Michaelis-Menten equation in a 58.6-fold increase in the import rate from 1/138 v_max_ to 101/238 v_max_ but also in a 101-fold dilution of the radioactive ^65^Zn so that the uptake velocity should be decreased to less than 58% of the initial value. While non-chased control cells continued to import ^65^Zn, chased cells exported ^65^Zn with an initial net efflux rate of 112 ^65^Zn cell^−1^ s^−1^, resulting in a decrease of the number of cell-bound ^65^Zn ions down to 37% of the content before the chase (Table 2). The total zinc content of zinc-replete cells had been 103,000 Zn per cell before the experiment and remained at this number during the uptake phase but 26% of the initially present zinc ions were exchanged for incoming zinc ions during the uptake period (Table 4). During the chase, this number increased to 246,000 Zn per cell due to the higher concentration of zinc ions in the environment but most of the zinc that had been imported during the uptake period was exported again (Table 4).

This demonstrated a continuous turnover of zinc ions in zinc-replete cells of *C. metallidurans*, meaning that zinc uptake and zinc efflux reactions were running simultaneously. A similar turnover was also visible in zinc- and metal-, meaning zinc-magnesium-, starved cells. Metal-starved AE104 cells contained a lower initial zinc content than zinc-replete cells, imported zinc with a seven times higher initial import rate. They reached an equilibrium of the zinc concentration already during the uptake period (Fig. 2) and subsequently exported ^65^Zn again even in the non-chased control while zinc-replete cells continued to accumulate the radioactive isotope in the control with a small rate of 14 ^65^Zn cell^−1^ s^−1^ compared to the initial 162 ^65^Zn cell^−1^ s^−1^ (Table 2). This means that the cells had been able to adjust the zinc import and export rates during the 20-min uptake period.

Regulation of the zinc uptake and efflux activities could have been done by flux control, for example, by binding of metal ions to the regulatory sites of the transporter (35, 36, 45, 46) or by interaction with other proteins, degradation of uptake or *de novo* synthesis of efflux systems. Expression of a gene for a copper-exporting P-type ATPase at 37°C in *Escherichia coli* needed just 2 min to reach an abundance peak for the transcript (47) and 10 min for a *czc* transcript at 30°C in *C. metallidurans* (48). Including the subsequent translation and membrane insertion, this would be just a sufficient time to explain an up-regulation of the efflux activities by protein synthesis. On the other hand, any flux control of the activities of import and export reactions would be a much quicker process and would allow a rapid adjustment of the turnover rate of zinc to changing zinc availability in the environment.

Although a flux control of the transport activities has a lower energetic cost compared to the synthesis and degradation of proteins, a constant turnover of cytoplasmic against environmental zinc is in fact a futile cycle. Efflux of zinc by the P-type ATPase ZntA is driven by ATP hydrolysis (19) and uptake for instance by uniport (5). Many uptake systems have a broad substrate range, for instance, ZupT (49, 50), CorA (51–53), and PitA as metal phosphate importer (54). Most transition metal cations have a similar ionic diameter. When the shell of water molecules has been removed, the radius of Co(II) and Zn(II) is 74 nm that of Mg(II) 65 nm (37). The electronic conformation of Zn(II) and Mg(II) is quite similar, with completely filled 3s and 3d orbitals in the case of Zn(II) and completely filled 2s and 2p orbitals for Mg(II) (55). Discrimination between Zn(II) and Mg(II) requires to explore the small differences in size, and the better ability of Zn(II) to form tetrahedral complexes.

A transport process that discriminates Zn(II) against Mg(II) by complex formation would be a slow process compared to a process that just selects sizes of divalent metal cations because the release of the initially bound cation during the subsequent transport reaction is an energetic barrier (5). Such discrimination can be easily performed by periplasmic metal-binding proteins of ABC transporters such as ZnuABC for Zn(II) (27, 32) or NikABC (56–58) for Ni(II). These highly substrate-specific import systems are absent in *C. metallidurans* so import of Mg(II) and transition metal cations depends on two P-type ATPases of the Mg/Ca group and secondary import systems (Fig. 1). To allow a high-rate import reaction by these transporters, their substrate discrimination does not allow the formation of tightly bound metal complexes at the substrate binding site but only gating mechanisms that are charge- and size-selective (59–61).

In the case of Mg(II), 12.5 million Mg(II) need to be imported during a generation time, under stress conditions even 40 million (Table S4), compared to 100,000 Zn(II) (Table 4) so that *C. metallidurans* needs 125 times more Mg(II) than Zn(II). The concentration of both metals in an ecosystem such as seawater is 55.5 mM Mg(II) and 153 nM Zn(II) (37), a factor of 363,000. Zinc availability in most environments is much lower than that of magnesium. This means that a high-rate uptake of Mg(II) with a broad substrate specificity automatically provides the cell also with divalent transition metal cations such as Zn(II), Co(II), and Ni(II). If this is not sufficient, many bacteria can produce ABC or ECF-ABC importers such as ZnuABC, NikABC, or CbiMNQO (32, 56, 58, 62–64).

The secondary transport systems such as CorA and Mg-importing P-type ATPases, thus provide Mg(II) to the *C. metallidurans* cell plus a bouquet of transition metal cations. In the second step, surplus metal cations are being removed again. This is done in the plasmid-free strain AE104 by P_IB2_-type exporters such as ZntA for Zn(II) and CadA for Cd(II) (20). Both proteins have a very similar affinity for Zn(II) and Cd(II) (19) but different MerR-type regulators control the expression of the respective genes, ZntR for *zntA* and CadR for *cadA* (7). This elegantly delegates the time-consuming process of substrate discrimination by complex formation to the regulatory proteins so that ZntA is only expressed at high internal zinc concentrations and CadA, when the Cd-exporting activity of ZntA is not sufficient to remove incoming cadmium. This was clearly demonstrated by the removal of cytoplasmic zinc when the cells were chased with cadmium (Table 3).

Acquisition of divalent metal cations is a very efficient process (5). The secondary import systems plus the P-type Mg importers MgtA and MgtB provide a broad range of metal cations and metal phosphates to the cell. If this is not sufficient for an individual component, in most bacteria, ABC-type importers with periplasmic substrate-binding proteins or ECF-ABC importers are being induced, in *C. metallidurans* just PstABC for phosphate. On the other hand, if too much of an individual metal arrives in the cell, efflux systems are up-regulated removing the specific metal cation again. Theoretically, this could be described as a flow equilibrium of import and export reactions, which adjusts the cytoplasmic availability of an individual metal cation and also the composition of the metal cation bouquet (40). The data presented here provide evidence that such a constant turnover of zinc ions is indeed occurring in *C. metallidurans*.

### Metal uptake, efflux, and buffering substances interact to generate the flow equilibrium

As could be expected, the turnover of zinc in *C. metallidurans* strain AE104 was affected in mutants with multiple deletions in the uptake systems ∆7 (*∆zupT ∆pitA ∆corA1 ∆corA2 ∆corA3 ∆zntB ∆hoxN*) and ∆9 (∆7 ∆*mgtA ∆mgtB*). ∆7 and ∆9 contained a lower initial zinc content ZP1 connected with a high initial uptake velocity v_up_(0) than all other strains (Tables 2 and 4). As already mentioned (30, 34), this unambiguously demonstrated the presence of at least one more uptake system “X” for zinc (Fig. 1). While zinc-magnesium-starved cells of ∆7 and ∆9 presented a very low amount of accumulated zinc during the uptake phase, zinc-replete cells of ∆9 but not ∆7 accumulated zinc with a higher rate than the parent; however, an equilibrium was reached after 15 min and the cells subsequently exported the metal again (Fig. 3A). This was a surprise. Search for “X” has been performed in the past by looking at ∆7 and ∆9 cells under metal-starvation conditions. Under these conditions, however, “X” was either down-regulated or on a low activity level.

In addition to the uptake systems, polyphosphate and glutathione also affected zinc uptake and efflux (Fig. 5). While zinc-replete mutant cells without polyphosphate and glutathione were not different from each other, deletion of *gshA* for the first step of glutathione decreased zinc uptake more than an interrupted polyphosphate synthesis in metal-starved cells. This demonstrated that components that should sequester zinc ions in the cytoplasm and subsequently decrease zinc availability in this compartment are also integral parts of zinc homeostasis, especially under metal-starvation conditions. The flow equilibrium of metal uptake and efflux reactions was buffered by cytoplasmic metal-binding components.

It could also be expected that efflux systems influenced the turnover of zinc because they performed the second part of the kinetical flow equilibrium of import and export reactions. Efflux of ^65^Zn after the uptake period was indeed on a lower level in the efflux mutants ∆e2 (∆*cadA ∆zntA*) and ∆e4 (∆e2 ∆*dmeF ∆fieF*) than in the parent strain AE104 (Fig. S6; Fig. 4; Table 2). The initial efflux rates v_eff_ decreased compared to the parent down to 26% in zinc-replete and 27% in metal-starved ∆e2 cells, and to 16% in zinc-replete and 9% in metal-starved ∆e4 cells (Table 2).

In zinc-replete ∆e2 and ∆e4 cells, the calculated C_max_ value of the uptake period was much higher than the measured C_20_ value (Table 2), indicating that strains without efflux systems had not reached an equilibrium during the uptake period. The C_max_/C_20_ value in zinc-replete cells was 5.5 in ∆e2 and 8.3 in ∆e4 and were the highest values measured in the experiments. This indicated that the activity of the efflux systems was essential to reach a flow equilibrium within 20 min. Other strains with high C_max_/C_20_ ratios in zinc-replete cells were ∆*ppk* (4.7) > ∆*gshA* = ∆*zupT* (2.7) >parent strain AE104 (2.2; Table 2). Polyphosphate, glutathione, and the zinc importer ZupT were also required to reach an equilibrium within the uptake period.

The data also demonstrated that the secondary efflux systems DmeF and FieF were involved in zinc efflux although their contribution to zinc resistance was neglectable (19). Moreover, reminiscent to “X,” still another zinc efflux system, might be present in *C. metallidurans* AE104, although the zinc resistance of the ∆e4 mutant is already down to an IC_50_ of 7.1 µM (19).

### Strain AE104 as plasmid-free mutant of *C. metallidurans* CH34 wild type

The two plasmids pMOL28 and pMOL30 of *C. metallidurans* CH34 wild type are crowded with metal resistance determinants (e.g., *czc, cnr, chr, cop, pbr, mer*) for cobalt, zinc, cadmium, nickel, copper, lead chromate, and mercury resistance (6, 18, 65–68). Among other genes for periplasmic components and two exporters of the inner membrane, the central product of *czc* is the RND-driven transenvelope pump CzcCBA, which exports Co(II), Zn(II), and Cd(II) from the periplasma across the outer membrane back to the outside (42–44, 69, 70). This adjusts the periplasmic metal concentration for a subsequent import into the cytoplasm and removes cations that just had been exported by inner membrane efflux systems, leading to a high level of zinc, cobalt, and cadmium resistance (18).

This convenient situation was over in the plasmid-free derivative *C. metallidurans* strain AE104 (18), resulting in decreased resistance to all the metals mentioned above. The difference between strains CH34 and AE104 was also evident in the pulse-chase experiments in zinc-replete cells of both strains. In the case of CH34, the cells are additionally pre-incubated in the presence of 100 µM Zn(II) to induce up-regulation of *czc* on plasmid pMOL28. While AE104 and non-induced CH34 cells possessed similar zinc contents at the beginning of the experiment, induced CH34 cells contained about threefold higher zinc content and a down-regulated initial uptake velocity v_up_(0) (Tables 2 and 4). Despite the similarities in the initial zinc content and v_up_(0) between AE104 and non-induced CH34 cells, AE104 imported C_20_ = 100,000 ^65^Zn ions during the uptake phase, CH34 cells under both conditions only 20,000 to 30,000. The CH34 wild-type cells chased with 1 mM Zn(II) were even able to keep the amount of accumulated zinc at a tolerable level, especially, when they were pre-incubated in the presence of 0.1 mM Zn(II). This demonstrated again the outstanding ability of *C. metallidurans* CH34 wild type to resist high concentrations of external zinc ions and to shield its cell against this metal.

## MATERIALS AND METHODS

### Bacterial strains and growth conditions

Plasmids and *C. metallidurans* strains (Table 1) in this study were all derivatives of the plasmid-free strain AE104 that lacks pMOL28 and pMOL30 (18), except of course the wild-type CH34. The ∆*ppk::lacZ* strain with *ppk* interrupted by *lacZ*-insertion (40) was produced before the experiment by conjugation of *E. coli* (pLO2::∆*ppk-lacZ*) and subsequent homologous recombination. Tris-buffered mineral salts medium (18) containing 2 g sodium gluconate/L (TMM) was used to cultivate these strains aerobically with shaking at 30°C. Since the purity of the mineral salts has been steadily improved, the concentration of contaminating zinc chloride and zinc added with the trace element solution SL6 (38) was no longer sufficient to supply sufficient zinc to the cells. Consequently, the zinc content of TMM was determined by ICP-MS (Inductively coupled plasma mass spectrometry) and subsequently adjusted to 200 nM by the addition of zinc chloride. This TMM was designated moderate zinc “mZn medium.” Moreover, zinc and metal starvation media were also used. A low zinc (lZn) medium was TMM without added trace element solution SL6 (38). A third medium was a low metal medium (lZn_lMg), TMM without SL6, and 100 µM magnesium chloride instead of 1 mM. Analytical grade salts of transition metal cations were used to prepare 1 M stock solutions, which were sterilized by filtration. Solid Tris-buffered media contained 20 g agar/L.

As determined by inductively coupled plasma mass spectrometry (ICP-MS), the metal contents of all media were 208 ± 15 µM calcium and 3.9 ± 0.2 µM iron (200 µM and 4.3 µM added, respectively). TMM, mZn, and lZn media contained 953 ± 86 µM magnesium (1 mM added) and medium lZn_lMg 85.2 ± 1.4 µM magnesium (0.1 mM added). The content of other metals in media TMM and mZn, which contained trace element solution SL6, were 48.9 ± 4.5 nM Mn(II), 86.3 ± 17.3 nM Co(II), 157 ± 19 nM Ni(II), 16.3 ± 14.0 nM Cu(II) (15.2 nM Mn, 84.1 nM Co, 8.42 nM Ni and 5.87 nM Cu, respectively, coming from a trace element solution), and in media lZn and lZn_lMg without SL6 34.6 ± 0.9 nM Mn(II), 4.6 ± 1.8 nM Co(II), 89.1 ± 2.5 nM Ni(II), and 14.3 ± 5.0 nM Cu(II) coming from contaminations so that the media lZn and lZn_lMg were actually low zinc and low cobalt media, whereas the contaminations were sufficient sources for manganese and nickel. Not-adjusted TMM contained 63.6 ± 9 nM zinc, adjusted mZn-TMM 200 nM. Media without SL6 contained 35.2 ± 30.4 nM zinc. Without SL6, the zinc content of the growth media varied strongly but were always in a range leading to zinc starvation conditions.

### Inductively coupled plasma mass spectrometry

For ICP-MS analysis, HNO3 (trace metal grade; Normatom/PROLABO) was added to the samples to a final concentration of 67% (wt/vol) and the mixture was mineralized at 70°C for 2 h. Samples were diluted to a final concentration of 2% (wt/vol) nitric acid. Indium and germanium were added as internal standards at a final concentration of 1 ppb and 10 ppb each. Elemental analysis was performed *via* ICP-MS using Cetac ASX-560 sampler (Teledyne, Cetac Technologies, Omaha, Nebraska), a MicroFlow PFA-100 nebulizer (Elemental Scientific, Mainz, Germany), and an ICAP-RQ ICP-MS instrument (Thermo Fisher Scientific, Bremen) operating with a collision cell and flow rates of 4.5 mL x min-1 of He/H_2_ [93%/7% (71)], with an Ar carrier flow rate of 0.76 L × min^−1^ and an Ar make-up flow rate at 15 L x min^−1^. An external calibration curve was recorded with ICP-multi-element standard solution XVI (Merck) in 2% (vol/vol) nitric acid. The sample was introduced *via* a peristaltic pump and analyzed for its metal content. For blank measurement and quality/quantity thresholds, calculations based on DIN32645 TMM were used. The results were calculated from the ppb data as atoms per cell as described (25).

### Pulse-chase experiments with radioactive ^65^Zn

Cells were incubated in TMM for 17 h at 30°C shaking at 200 rpm, diluted 20-fold into a second pre-culture in the medium that was used for the subsequent main culture (TMM, mZn, lZn, lZn_lMg), and incubated with shaking at 30°C for 24 h. Cells were diluted 50-fold into the main culture, which was incubated with shaking at 30°C at 200 rpm until a turbidity of 150 Klett units was reached (mid-exponential phase of growth). The cells were harvested by centrifugation at 4°C, washed in the same volume of 10 mM TrisHCl (pH 7), suspended in the same volume of 10 mM TrisHCl (pH 7), and kept on ice until needed during the same day. For the experiments, sodium gluconate was added to 6 mL of the cell suspension to a final concentration of 2 g/L directly before the start to provide energy to the cells. At t = 0, radioactive ^65^Zn was added to the cell suspension to a final concentration of 1 µM Zn(II) and 60 nCi/mL. The ^65^ZnCl_2_ was supplied by POLATOM (certificate 022–106722-03622-0001).

The cells were incubated with shaking at 30°C. At 0.25, 5, 10, and 15 min, samples of 500 µL were removed, and filtered through a membrane filter (0.2 µm pore size, Whatman cellulose nitrate membrane filters, Cytiva) using a vacuum-driven uptake apparatus. The samples were rapidly washed twice with 5 mL of 50 mM TrisHCl (pH 7) containing 50 mM EDTA. The activity was counted in a Liquid Scintillation Counter (PerkinElmer Tri-Carb 2810 TR) using Ultima Gold (PerkinElmer). The samples were counted twice for 2 min in a window from 0 to 200 keV. In some experiments, ^63^Ni (Amersham Biosciences) was used instead of ^65^Zn.

For the chase, non-radioactive zinc was added at t = 20 min to a final concentration of 100 µM or 1 mM. In some experiments, other transition metal chlorides or EDTA were used. Incubation was continued with shaking at 30°C and samples were removed at 20.25, 25, 30, 35, and 40 min. They were treated and analyzed as described above for the samples of the uptake period.

A sample of 100 µL was counted to determine the total radioactivity of the ^65^Zn in the cell suspension used for the pulse-chase experiment. From this value, the mol zinc per cpm ratio was derived. For each time sample, the mean value and technical deviation of the two 2-min counts were calculated. Two zero controls were subtracted, one for the background radioactivity at the time of the experiment and one for the chemical adsorption of ^65^Zn by the membrane filter. The resulting value was multiplied by the mol/cpm ration of the respective experiment to give the mol ^65^Zn per 500 µL time sample. The actual cell number in the sample had been determined *via* an equilibration curve for the turbidity at 600 nm so that the mol ^65^Zn per cell and subsequently the number of the ^65^Zn atoms per cell could be calculated.

All experiments were performed at least three times. For each experiment, the zinc content per cell at 7.5 min was calculated from the 5 min and 10 min values. This value was used to correct the number of atoms per cell for all experiments involving the same mutant and the same growth condition. Experiments with large correction factors were removed and the respective experiment was repeated. For each strain and condition, the mean values and deviations of the ^65^Zn atoms per cell were finally calculated. This value was designated as the cellular metal content C(t) for the respective mutant and growth condition.

Pulse-chase with ^65^Zn measured: (i) the initial zinc uptake velocity v_up_(0) at t = 0; (ii) the cellular ^65^Zn content C_20_ at the end of the uptake period (time point represented in Fig. 2 by the horizontal bar); (iii) the extrapolated maximum zinc content after the uptake period C_max_; (iv) the efflux velocity v_eff_ at the beginning of the chase at 20 min; (v) the corresponding initial zinc content C_0_ used to calculate v_eff_; and (vi) the final zinc content C_40_ at the end of the chase period (Fig. 2, t = 40 min). To obtain these data, the uptake phase up to 20 min of the pulse-chase experiment was adapted to the equation C(t) =C_max_^.^ t/(K_t_ +t) using the Lineweaver-Burk-like plot 1/C(t) = 1/C_max_ + (K_t_/C_max_^.^ 1/t). The first deviation by time of the equation C(t) =C_max_^.^ t/(K_t_ + t) was dC(t)/dt = C_max_^.^ K_t_/(K_t_ + t)^2^. At t = 0, this gave the initial uptake rate v_up_(0) = C_max_/K_t_. After the chase after 20 min, the cell-bound zinc content was modeled by the decay function C(t) =C_o_^.^ e^(−τ ^.^ t) using the plot ln C(t) =ln C_o_ −τ ^.^ t. The first deviation by time of the equation C(t) =C_o_^.^ e^(−τ ^.^ t) was dC(t)/dt = −τ ^. .^C_o_^.^ e^(−τ ^.^ t). And at t = 0, this value was the initial net efflux rate v_eff_(0) = −τ ^.^ C_o_. In contrast to the initial uptake rate that was no net rate because the cells did not contain ^65^Zn at t = 0, v_eff_(0) was a net rate and the result of the real efflux rate after chase minus the rate of ^65^Zn re-import at this time.

### Experiments with stable ^67^Zn

Stable enriched ^67^Zn was employed to determine (vii) the resident zinc pool (ZP) ZP1 at the beginning of the experiment; (viii) the zinc pools ZP1 and ZP2 after the uptake period; and (ix) finally these pools ZP1 and ZP2 after the chase period. The cell suspensions were prepared in the respective media as described for the pulse-chase experiments above; however, the respective growth medium was used instead of the uptake buffer for these experiments. After a zero sample had been removed for the ICP-MS analysis, isotope-enriched ^67^Zn(II) was added to a final concentration of 1 µM. Incubation was continued with shaking for 20 min, a sample was removed and the remaining cells chased with non-enriched Zn(II) added at a final concentration of 100 µM or 1 mM. In some experiments, other metal chlorides or EDTA were used for the chase. Incubation was continued for 20 min with shaking at 30°C and the third sample was removed. The cells in the respective samples were harvested by centrifugation, washed twice with 50 mM TrisHCl buffer (pH 7.0) containing 50 mM EDTA at 0°C, suspended in 50 mM TrisHCl buffer (pH 7.0), and mineralized for the subsequent ICP-MS analysis. The ^67^Zn (94% ^67^Zn) was provided as metal from Nakima Ltd (Savyon, Israel) and oxidized using HCl on ice. The zinc content was verified by ICP-MS.

For the calculation of different zinc pools in the cells, the ratio of ^67^Zn in the isotope-enriched zinc solution (94%) and non-enriched “usual” zinc [4.1%; (37)] was used. The ICP-MS measurement calculates the quantity of an element from that of its isotopes, thereby correcting for the % of the natural abundance of the respective isotope. The zinc pool 1 (ZP1) was defined as the cellular zinc pool before the addition of isotope-enriched ^67^Zn and was equal to the ^64^Zn ICP-MS result [natural abundance 48.6%; (37)]. Similar results were obtained using ^66^Zn (27.9%) instead of ^65^Zn. Zinc pool 2 (ZP2) was the zinc pool after incubation of the cells with ^67^Zn. ZP2 was the ^67^Zn value coming from the ICP-MS (corrected for a natural abundance of 4.1%) minus the ^64^Zn value (0.75% in the ^67^Zn-enriched zinc solution) and the result was divided by 22.2346.

### Statistics

Students’ *t*-test was used but in most cases the distance (D) value, D, has been used several times previously for such analyses (34, 72, 73). It is a simple, more useful value than Student’s *t*-test because non-intersecting deviation bars of two values (D > 1) for three repeats always means a statistically relevant (≥ 95%) difference provided the deviations are within a similar range. At *n* = 4, significance is ≥97.5%, *n* = 5 ≥ 99% (significant), and *n* = 8 ≥ 99.9% (highly significant).

## SUPPLEMENTAL MATERIAL

Supplemental material jb.00080-24-s0001.pdf Tables S1 to S5; Figures S1 to S7.

ASM does not own the copyrights to Supplemental Material that may be linked to, or accessed through, an article. The authors have granted ASM a non-exclusive, world-wide license to publish the Supplemental Material files. Please contact the corresponding author directly for reuse.
