# Supplementary material for: A flow equilibrium of zinc in cells of Cupriavidus metallidurans
Source: J Bacteriol. 2024 Apr 25;206(5):e00080-24. doi: 10.1128/jb.00080-24 (PMC11112998; doi:10.1128/jb.00080-24)
Supplement: Supplemental material — Tables S1 to S5; Figures S1 to S7. [file jb.00080-24-s0001.pdf]

## Supplementary Material

Overview

SUPPLEMENTARY TABLE S1: BACTERIAL STRAINS ........ 1
PROOF OF CONCEPT ........ 2
*C. METALLIDURANS* AE104 AS REFERENCE POINT: CHASE WITH ZINC AND OTHER METALS ........ 5
ROLE OF UPTAKE SYSTEMS. ........ 12
ROLE OF EFFLUX SYSTEMS. ........ 13
*C. METALLIDURANS* CH34 WILD TYPE ........ 14
LITERATURE OF THE SUPPLEMENT ........ 15

## Supplementary Table S1: Bacterial strains

| Strain | Relevant markers | Reference |
|---|---|---|
| | *Cupriavidus metallidurans* | |
| CH34 | pMOL28, pMOL30 | (1) |
| AE104 | no plasmid | (1) |
| DN861 | AE104 ∆*gshA* | (2) |
| DN600 | AE104 ∆e2, (∆*zntA* ∆*cadA)* | (3) |
| DN578 | AE104 ∆e4, (∆*zntA* ∆*cadA* ∆*dmeF* ∆*fieF*) | (3) |
| DN515 | AE104 ∆*zupT* | (4) |
| DN680 | AE104 ∆7, (∆*corA1* ∆*corA2* ∆*corA3* ∆*zupT* ∆*pitA,* ∆*zntB* ∆*hoxN*) | (5) |
| DN784 | AE104 ∆9, (∆7 ∆*mgtA* ∆*mgtB*) | (6) |
| | *Escherichia coli* | |
| ECB180 | pLO2::∆*ppk-lacZ* (for construction of AE104 ∆*ppk-lacZ*) | (7) |

## Proof of concept

**Supplementary Table S2. Zinc content of *C. metallidurans* strain AE104 in TMM medium**

| | not $^{67}$Zn-enriched, $10^3$ Zn per cell | | | $^{67}$Zn-enriched, $10^3$ Zn per cell | | |
|---|---|---|---|---|---|---|
| Zn(II), (µM) | ZP1 | ZP2 | ZP1+ZP2 | ZP1 | ZP2 | ZP1+ZP2 |
| mZn-TMM | | | | | | |
| 0 | 101±4 | <0 | 101±4 | 95±0 | <0 | 95±0 |
| 1 | 127.5±6.8 | 0.9±1.4; 0.7% | 128±8 | 52.7±2.1 | 55.9±1.4; 51.5% | 109±4 |
| 10 | 155±1 | <0 | 155±1 | 40±2 | 100±3; 71.4% | 140±5 |
| 100 | 241±9 | 0.7±0.3; 0.3% | 242±10 | 42±2 | 135±11; 76.1% | 178±13 |
| lZn-TMM without SL6 | | | | | | |
| 0 | 7.0±1.2 | 0.0±0.0; 0.4% | 7.1±1.2 | 12.7±5.1 | 0.1±0.0; 0.8% | 12.8±5.1 |
| 1 | 113.2±7.7 | 0.1±0.1; 0.1% | 113±8 | 11.5±2.8 | 94.5±3.1; 89.2% | 107±6 |
| 10 | 138±9 | <0 | 138±9 | 7±2 | 109±6; 93.8% | 116±8 |
| 100 | 186±18 | <0 | 186±18 | 10±0 | 144±4; 93.3% | 155±5 |
| lZn_lMg-TMM | | | | | | |
| 0 | 9.3±3.6 | 0.1±+0.1; 1.3% | 9.5±3.5 | 11.3±6.2 | 0.1±0.0; 0.5% | 11.4±6.1 |
| 1 | 119.3±7.4 | 0.1±0.0; 0.1% | 119±7 | 9.2±3.5 | 97.7±0.1; 91.4% | 107±4 |
| 10 | 162±13 | 1.0±1.3; 0.6% | 163±14 | 8±2 | 121±12; 94.2% | 129±14 |
| 100 | 212±13 | <0 | 211±12 | 9±1 | 173±1; 95.2% | 181±2 |

*C. metallidurans* AE104 was cultivated in TMM medium adjusted to 200 nM Zn(II), TMM without trace element solution SL6 but 1 mM Mg(II), or low zinc low magnesium TMM without SL6 and 0.1 mM Mg(II). The initial medium contained zinc with the natural isotope composition. After the turbidity had reached 100 Klett units, stable $^{67}$Zn(II) or Zn(II) with a natural isotope composition was added at the indicated concentration. Incubation was further continued with shaking to a turbidity of 150 Klett units and the metal content was determined by ICP+MS. The different zinc pools were determined by the $^{64}$Zn content (ZP1) and the over-calculation of the overall zinc content stemming from the $^{67}$Zn measurement after addition of the $^{67}$Zn-enriched zinc solution, ZP2 =($^{67}$Zn-$^{64}$Zn)/ 23.2346. The total zinc content is ZPt = ZP1+ZP2. Moreover, the percentage of ZP2 of the total zinc content is also indicated.

**Supplementary Table S3. Metal content of *C. metallidurans* strain AE104 in TMM adjusted to 200 nM Zn(II).**

| Addition | Atoms per cell | | | | | | |
|---|---|---|---|---|---|---|---|
| Medium | Mg, $10^6$ | Ca, $10^3$ | Fe, $10^3$ | Mn, $10^3$ | Co, $10^3$ | Ni, $10^3$ | Cu, $10^3$ |
| <u>all Zn isotopes</u> | | | | | | | |
| mZn (200 mM) | | | | | | | |
| 0 µM Zn | 13.4±0.7 | 251±9 | 998±4 | 0.21±0.09 | 5.3±0.4 | 4.2±0.2 | 5.6±0.4 |
| 1 µM Zn | 14.8±0.6 | 231±27 | 1082±14 | 0.19±0.05 | 4.7±0.3 | 4.4±0.2 | 4.7±0.2 |
| 10 µM Zn | 14.1±0.3 | **197±18** | 1059±33 | 0.16±0.03 | 4.4±0.2 | 4.0±0.0 | 4.7±0.3 |
| 100 µM Zn | **17.6±0.4** | **139±8** | 834±37 | 0.33±0.30 | 4.4±0.0 | 3.4±0.0 | 5.1±0.2 |
| lZn | | | | | | | |
| 0 µM Zn | 13.3±0.4 | 193±43 | 1003±40 | 0.33±0.02 | 0.3±0.0 | 4.2±0.1 | 2.8±0.6 |
| 1 µM Zn | 13.0±0.5 | 277±98 | 850±52 | 0.26±0.11 | 0.2±0.0 | 3.3±0.2 | 5.3±3.4 |
| 10 µM Zn | 13.9±0.6 | 187±48 | 840±45 | **0.18±0.07** | 0.2±0.0 | 3.1±0.2 | 2.4±0.2 |
| 100 µM Zn | **16.6±0.7** | 142±43 | **709±14** | **0.12±0.01** | 0.2±0.0 | 2.6±0.1 | 2.8±0.5 |
| lZn_lMg | | | | | | | |
| 0 µM Zn | 13.9±0.5 | 240±44 | 1051±32 | 0.43±0.04 | 0.2±0.0 | 6.0±0.4 | 3.1±1.3 |
| 1 µM Zn | 13.3±0.5 | 237±29 | 897±12 | **0.72±0.47** | 0.2±0.0 | 5.4±0.4 | 2.5±1.4 |
| 10 µM Zn | 13.9±0.5 | 223±16 | **802±35** | **0.15±0.03** | 0.3±0.0 | 4.9±0.3 | 2.1±0.8 |
| 100 µM Zn | 13.8±0.7 | **147±22** | **692±48** | 0.37±0.18 | 0.2±0.0 | 4.9±0.4 | 3.9±1.3 |
| <u>$^{67}$Zn+enriched</u> | | | | | | | |
| mZn | | | | | | | |
| 0 µM Zn | 12.2±0.0 | 281±17 | 861±13 | 0.37±0.03 | 4.0±0.7 | 4.6±0.2 | 4.4±0.2 |
| 1 µM Zn | 12.2±0.2 | 293±5 | 867±14 | 0.26±0.04 | 3.2±0.4 | 4.2±0.1 | 4.2±0.4 |
| 10 µM Zn | 12.7±0.4 | 251±15 | 867±57 | **0.19±0.05** | 3.0±0.3 | 3.8±0.3 | 4.0±0.2 |
| 100 µM Zn | **15.3±0.2** | **183±8** | **741±53** | 0.42±0.34 | 3.3±0.5 | 3.3±0.1 | 6.2±0.1 |
| lZn | | | | | | | |
| 0 µM Zn | 11.9±0.6 | 238±20 | 921±53 | 0.46±0.19 | 0.2±0.0 | 4.0±0.2 | 4.0±1.5 |
| 1 µM Zn | 12.6±0.4 | 259±5 | 813±15 | **0.17±0.03** | 0.2±0.0 | 3.7±0.3 | 2.6±0.4 |
| 10 µM Zn | 12.7±0.5 | 208±12 | 719±10 | **0.10±0.02** | 0.2±0.0 | 2.9±0.0 | 2.6±0.4 |
| 100 µM Zn | **15.6±0.5** | 190±34 | **637±13** | **0.14±0.02** | 0.3±0.0 | 2.9±0.5 | 4.8±0.3 |
| lZn_lMg | | | | | | | |
| 0 µM Zn | 12.4±0.5 | 266±30 | 932±36 | 0.39±0.05 | 0.2±0.0 | 5.6±0.3 | 1.6±0.4 |
| 1 µM Zn | 12.2±0.2 | 273±24 | 792±53 | 0.29±0.12 | 0.2±0.0 | 5.1±0.4 | 2.8±0.9 |
| 10 µM Zn | 12.0±0.2 | 260±38 | **725±31** | 0.18±0.06 | 0.2±0.0 | 4.7±0.2 | 1.5±0.4 |
| 100 µM Zn | 13.5±0.4 | 212±5 | **609±66** | 0.15±0.02 | 0.2±0.0 | 4.7±0.1 | 3.5±0.4 |

*C. metallidurans* AE104 was cultivated in mZn-TMM medium adjusted to a zinc concentration of 200 nM up to a turbidity of 100 Klett units, Zn(II) was added at the indicated concentration. Incubation was continued with shaking to a turbidity of 150 Klett units and the metal content was determined by ICP+MS. Bold-faced letters indicate a significant (D > 1) decrease by 20% compared to the cells cultivated without zinc in the same medium (first line of the respective row).

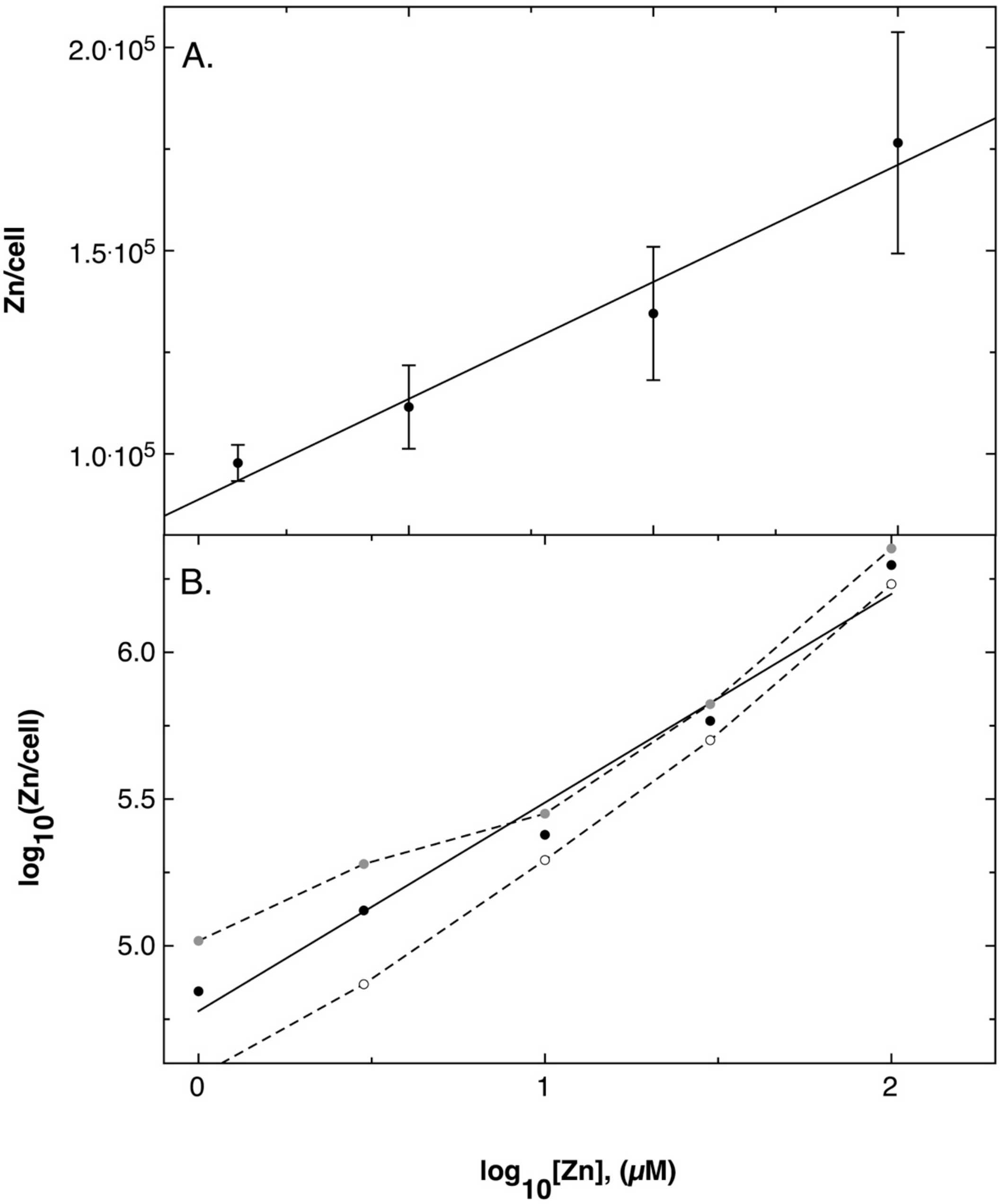


**Supplementary Figure S1. The cellular zinc content of *C. metallidurans* depends on the zinc content of the growth medium**. The mean values of the zinc content of cells cultivated in mZn-TMM adjusted to 200 nM Zn(II), or in adjusted or not-adjusted TMM in the presence of 1 µM, 10 µM or 100 µM $^{67}Zn$ as determined by ICP-MS or using radioactive $^{65}Zn$ were plotted against the zinc content of the growth medium. The resulting half-logarithmical function (Panel A, regression coefficient 98.4%) indicated a cellular zinc content at 1 µM Zn(II) of 113,530±4,310 Zn ions/cell and an increase of 28,796±3,680 Zn ions/cell with every 10-fold increase of the zinc content of the medium. For Panel B, the double-exponential plot was $log_{10}$(Zn/cell) = 4.7775±0.07726 + 0.7106±0.063274*$log_{10}$ (µM zinc), the regression coefficient was 98.8%. Deviation bars are only given for the half-exponential plot. For the double-exponential plot, the maximum values (grey dots) and minimal values (white dots) are indicated instead, in addition to the mean values (black dots)

**_C. metallidurans_ AE104 as reference point: chase with zinc and other metals**

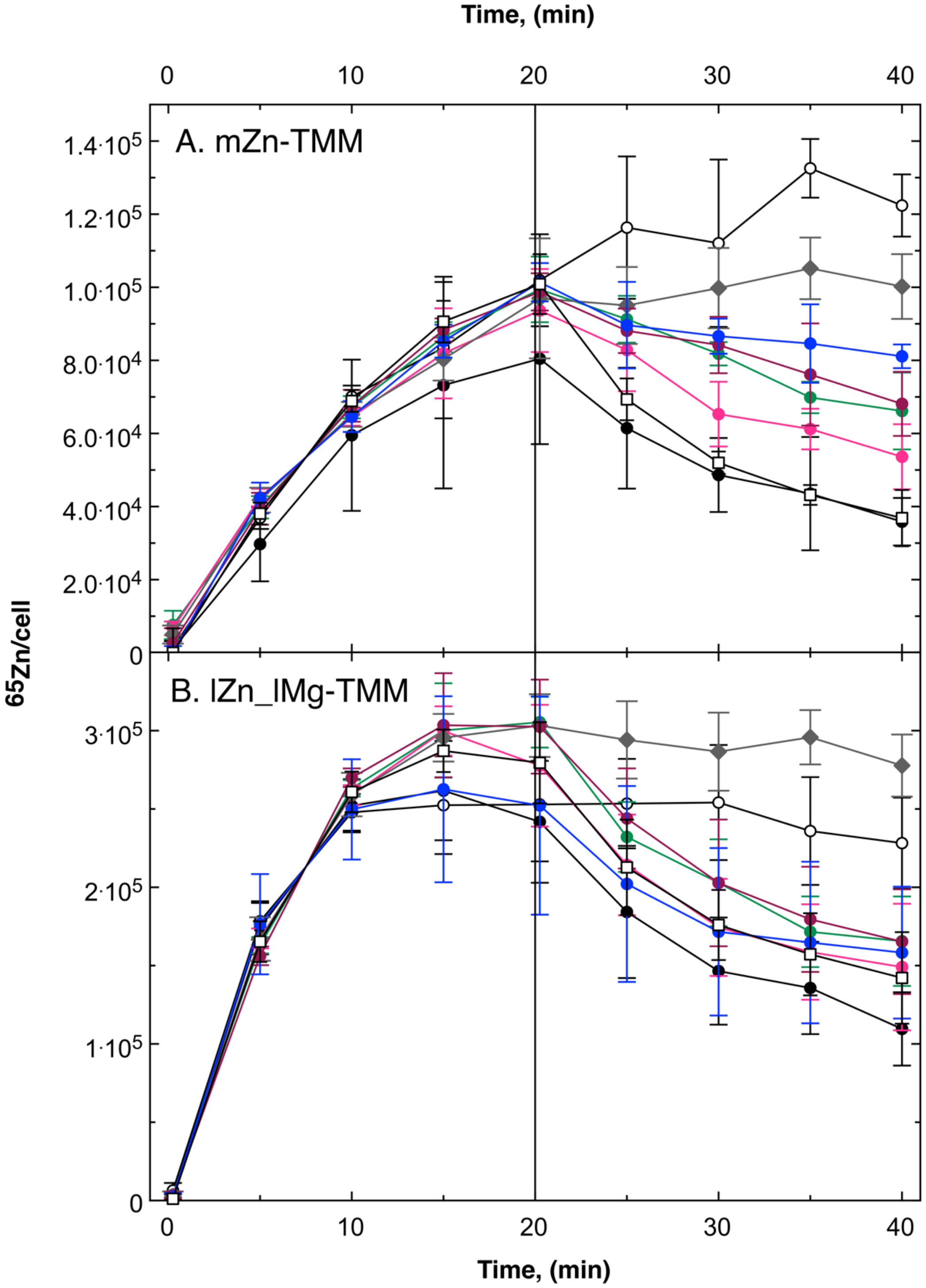


**Supplementary Figure S2. Pulse-chase-experiment with *C. metallidurans* strain AE104, zinc and other metal cations.** The cells were cultivated in TMM containing 200 nM Zn(II) (Panel A, mZn-TMM) or no added Zn(II) and 0.1 mM Mg(II) (Panel B, lZn_lMg, 35.2±30.4 nM Zn(II)) as described in Fig. 2. Pulse at t = 0 with 1 µM $^{65}$Zn(II), chase at t = 20 min with 100 µM non-radioactive Zn(II) (black circles), EDTA (open squares), Ni(II) (green circles), Co(II) (red circles), Cd(II) (grey diamonds), Mn(II) (brown circles), Mg(II) (blue circles) or not chased (open circles)

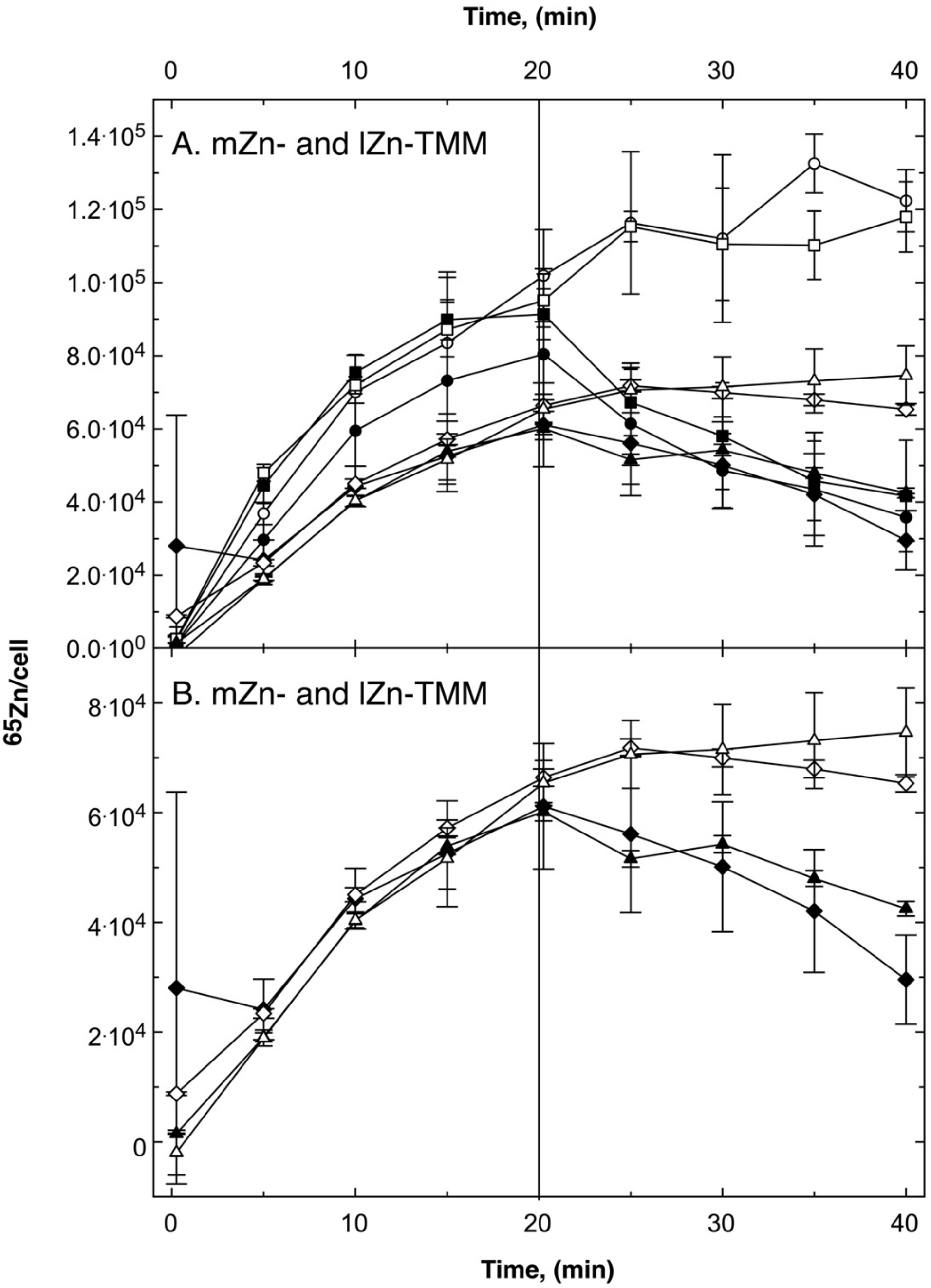


**Supplementary Figure S3. Pulse-chase-experiment with *C. metallidurans* strains AE104, ∆*zupT* and zinc.** Cells of strain AE104 (circles and squares) and its ∆*zupT* mutant (diamonds and triangles) were cultivated in TMM containing 200 nM Zn(II) (mZn-TMM, circles and diamonds) or no added Zn(II) and 1 mM Mg(II) (lZn, 35.2±30.4 nM Zn(II), squares and triangles) as described in Fig. 2. Pulse at t = 0 with 1 µM $^{65}$Zn(II), chase at t = 20 min with 100 µM non-radioactive Zn(II) (black symbols), or not chased (open symbols). Panel B contains the data from Panel A without those from strain AE104.

## Supplementary Table S4. Other metals[a]

| Strain/Condition | | $10^6$ Mg | $10^3$ Ca | Mn | $10^3$ Fe | $10^3$ Co | $10^3$ Ni | $10^3$ Cu |
|---|---|---|---|---|---|---|---|---|
| AE104 mZn | Atoms per cell | 12.5±0.9 | 258±55 | 575±306 | 1007±86 | 9.73±1.06 | 5.97±1.77 | 3.90±0.34 |
| | | ratio of the value of strain AE104 in mZn medium | | | | | | |
| <u>Moderate Zn</u> | | Mg ratio | Ca ratio | Mn ratio | Fe ratio | Co ratio | Ni ratio | Cu ratio |
| AE104 | Initial content | 1.00±0.07 | 1.00±0.21 | 1.00±0.09 | 1.00±0.53 | 1.00±0.11 | 1.00±0.30 | 1.00±0.09 |
| | Uptake | 1.01±0.02 | 0.94±0.24 | 0.98±0.04 | 0.65±0.40 | 0.92±0.13 | 0.86±0.12 | 0.89±0.04 |
| | Chase | 1.01±0.10 | 0.87±0.19 | 0.93±0.11 | ***0.48±0.28*** | 0.86±0.15 | 0.67±0.04 | 1.16±0.08 |
| | with 1 mM | 0.86±0.05 | 1.48±0.22 | 0.93±0.07 | 0.99±0.70 | 0.85±0.14 | 0.79±0.01 | 1.18±0.07 |
| CH34 | Initial content | 0.83±0.05 | 1.05±0.02 | 0.59±0.18 | 0.83±0.01 | ***0.27±0.02*** | 1.08±0.09 | 0.98±0.09 |
| | Uptake | 0.78±0.07 | 0.96±0.08 | 0.46±0.07 | 0.83±0.03 | ***0.25±0.02*** | 1.01±0.15 | 0.93±0.09 |
| | Chase | 0.84±0.02 | 0.96±0.08 | 0.32±0.02 | 0.75±0.01 | ***0.23±0.02*** | 0.88±0.12 | 0.86±0.05 |
| | with 1 mM | 0.70±0.01 | 1.04±0.00 | 0.28±0.02 | 0.73±0.03 | ***0.22±0.02*** | 0.81±0.11 | 0.95±0.05 |
| CH34 M1 | Initial content | 0.86±0.06 | 1.05±0.00 | 0.44±0.28 | 0.92±0.03 | ***0.07±0.00*** | 1.25±0.23 | 1.27±0.17 |
| + 0.1 mM Zn(II) | Uptake | 0.84±0.06 | 1.05±0.09 | 0.24±0.07 | 0.90±0.04 | ***0.06±0.00*** | 1.07±0.13 | 1.17±0.13 |
| | Chase | 0.77±0.09 | 1.06±0.05 | 0.20±0.08 | 0.77±0.02 | ***0.05±0.00*** | 1.00±0.04 | 0.93±0.01 |
| | with 1 mM | 0.84±0.03 | 0.89±0.05 | 0.11±0.02 | 0.76±0.02 | ***0.05±0.00*** | 0.90±0.10 | 0.98±0.05 |
| Δ*ppk* | Initial content | 0.79±0.04 | 1.01±0.12 | 0.31±0.04 | 0.92±0.01 | 1.12±0.47 | 0.88±0.27 | 1.00±0.09 |
| | Uptake | 0.76±0.06 | 1.00±0.07 | 0.23±0.01 | 0.83±0.01 | 0.99±0.37 | 0.68±0.09 | 0.82±0.03 |
| | Chase | 0.77±0.05 | 0.83±0.05 | 0.27±0.10 | 0.81±0.04 | 0.94±0.35 | ***0.60±0.01*** | 0.82±0.02 |
| Δ*gshA* | Initial content | 0.93±0.03 | 0.97±0.07 | 0.30±0.07 | ***0.62±0.00*** | ***0.50±0.05*** | ***0.52±0.09*** | 1.03±0.10 |
| | Uptake | 0.87±0.04 | 0.87±0.06 | 0.36±0.03 | ***0.59±0.01*** | ***0.47±0.04*** | ***0.38±0.04*** | 0.89±0.08 |
| | Chase | 0.82±0.05 | 0.75±0.03 | 0.14±0.04 | ***0.53±0.02*** | ***0.44±0.05*** | ***0.28±0.08*** | 0.82±0.06 |
| Δe2 | Initial content | 0.95±0.11 | 1.21±0.19 | 0.28±0.06 | 0.78±0.04 | ***0.44±0.04*** | ***0.30±0.03*** | 0.94±0.07 |
| | Uptake | 0.92±0.04 | 0.94±0.02 | 0.25±0.07 | 0.73±0.01 | ***0.39±0.04*** | ***0.18±0.04*** | 0.84±0.04 |
| | Chase | 0.76±0.05 | 0.81±0.06 | 0.07±0.01 | ***0.66±0.01*** | ***0.37±0.02*** | ***0.11±0.00*** | 0.80±0.04 |

| Content ratio | | Mg ratio | Ca ratio | Mn ratio | Fe ratio | Co ratio | Ni ratio | Cu ratio |
|---|---|---|---|---|---|---|---|---|
| Δe4 | Initial content | **1.76±0.06** | 0.96±0.07 | 0.22±0.02 | 0.75±0.03 | 0.94±0.09 | ***0.26±0.01*** | 0.82±0.02 |
| | Uptake | **1.91±0.15** | 0.94±0.03 | 0.22±0.04 | 0.79±0.06 | 0.92±0.08 | ***0.20±0.02*** | 0.77±0.03 |
| | Chase | **1.88±0.04** | 0.86±0.08 | 0.22±0.14 | 0.71±0.07 | 0.83±0.11 | ***0.17±0.03*** | 0.76±0.07 |
| Δ*zupT* | Initial content | 0.79±0.01 | 0.92±0.02 | 0.23±0.05 | 0.71±0.01 | ***0.53±0.02*** | ***0.33±0.02*** | 0.87±0.06 |
| | Uptake | 0.78±0.06 | 0.86±0.06 | 0.20±0.02 | 0.71±0.05 | ***0.52±0.04*** | ***0.23±0.03*** | 0.80±0.02 |
| | Chase | 0.70±0.01 | 0.75±0.04 | 0.10±0.04 | ***0.64±0.00*** | ***0.46±0.01*** | ***0.15±0.01*** | 0.82±0.13 |
| Δ7 | Initial content | **2.69±0.08** | 1.12±0.15 | 1.12±0.41 | 0.74±0.03 | ***0.63±0.04*** | ***0.49±0.10*** | 1.14±0.38 |
| | Uptake | **2.64±0.09** | 0.95±0.05 | 0.97±0.28 | 0.70±0.01 | ***0.59±0.02*** | ***0.29±0.01*** | 1.03±0.30 |
| | Chase | **2.89±0.13** | 0.85±0.05 | 0.79±0.25 | 0.68±0.02 | ***0.58±0.03*** | ***0.52±0.37*** | 1.03±0.28 |
| Δ9 | Initial content | **2.63±0.13** | 1.04±0.18 | 1.91±1.15 | 0.72±0.04 | ***0.66±0.03*** | ***0.53±0.11*** | 1.07±0.28 |
| | Uptake | **2.49±0.17** | 0.84±0.07 | 1.53±0.87 | ***0.64±0.04*** | ***0.59±0.01*** | ***0.31±0.03*** | 0.88±0.26 |
| | Chase | **2.89±0.12** | 0.75±0.01 | 1.33±0.83 | 0.68±0.01 | ***0.62±0.02*** | ***0.29±0.03*** | 0.93±0.27 |
| Low zinc medium | | | | | | | | |
| AE104 | Initial content | 1.05±0.03 | 0.81±0.09 | 0.95±0.04 | ***0.48±0.05*** | *0.02±0.00* | 0.80±0.07 | *0.55±0.08* |
| | Uptake | 1.07±0.01 | 0.77±0.13 | 0.92±0.01 | 1.42±0.74 | *0.01±0.00* | 0.83±0.03 | *0.57±0.07* |
| | Chase | 1.06±0.08 | 0.66±0.18 | 0.85±0.07 | ***0.23±0.04*** | *0.01±0.00* | 0.81±0.13 | *0.71±0.04* |
| | with 1 mM | 0.72±0.05 | 2.34±0.19 | 0.86±0.05 | ***0.52±0.12*** | *0.01±0.00* | 0.99±0.27 | *0.74±0.08* |
| Δ*zupT* | Initial content | 0.79±0.03 | 0.87±0.06 | 0.29±0.03 | 0.79±0.04 | *0.02±0.00* | ***0.21±0.02*** | *0.35±0.10* |
| | Uptake | 0.78±0.01 | **1.98±1.43** | 0.29±0.07 | 0.74±0.02 | *0.01±0.00* | ***0.15±0.01*** | *0.32±0.12* |
| | Chase | 0.76±0.03 | 0.78±0.08 | 0.22±0.07 | 0.71±0.03 | *0.01±0.00* | ***0.17±0.03*** | *0.42±0.17* |

Continued

| Content ratio | | Mg ratio | Ca ratio | Mn ratio | Fe ratio | Co ratio | Ni ratio | Cu ratio |
|---|---|---|---|---|---|---|---|---|
| Low Zn, low Mg | | | | | | | | |
| AE104 | Initial content | 1.03±0.02 | 0.91±0.25 | 0.95±0.01 | ***0.56±0.05*** | *0.01±0.00* | 1.14±0.10 | *0.26±0.04* |
| | Uptake | 1.06±0.01 | 0.83±0.11 | 0.90±0.02 | ***0.45±0.05*** | *0.01±0.00* | 1.03±0.08 | *0.24±0.03* |
| | Chase | 1.05±0.07 | 0.79±0.26 | 0.88±0.06 | ***0.34±0.04*** | *0.01±0.00* | 0.98±0.04 | *0.47±0.13* |
| | with 1 mM | ***0.66±0.04*** | 2.27±0.35 | 0.88±0.03 | ***0.58±0.09*** | *0.01±0.00* | 1.18±0.27 | *0.48±0.06* |
| Δ*ppk* | Initial content | 0.80±0.07 | 1.01±0.09 | 0.63±0.09 | 0.91±0.06 | *0.01±0.00* | 1.05±0.14 | *0.24±0.06* |
| | Uptake | 0.77±0.02 | 0.94±0.06 | 0.82±0.29 | 0.83±0.04 | *0.02±0.00* | **2.15±1.49** | *0.23±0.06* |
| | Chase | 0.75±0.03 | 0.71±0.04 | 0.30±0.02 | 0.77±0.03 | *0.01±0.00* | **1.60±1.16** | *0.25±0.03* |
| Δ*gshA* | Initial content | 0.96±0.14 | 0.88±0.10 | 0.57±0.05 | 0.68±0.04 | *0.02±0.00* | 0.90±0.06 | *0.27±0.04* |
| | Uptake | 0.96±0.05 | 0.80±0.07 | 0.43±0.04 | ***0.60±0.03*** | *0.02±0.01* | ***0.66±0.10*** | *0.23±0.04* |
| | Chase | 0.95±0.01 | 0.81±0.16 | 0.28±0.06 | ***0.58±0.01*** | *0.01±0.00* | ***0.51±0.03*** | *0.26±0.06* |
| Δe2 | Initial content | 0.98±0.06 | 0.84±0.07 | 0.50±0.08 | 0.82±0.05 | *0.01±0.00* | ***0.53±0.03*** | *0.20±0.03* |
| | Uptake | 0.94±0.03 | 0.76±0.07 | 0.39±0.07 | 0.73±0.04 | *0.01±0.00* | ***0.39±0.03*** | *0.17±0.02* |
| | Chase | 0.91±0.05 | 0.74±0.05 | 0.23±0.04 | 0.69±0.05 | *0.01±0.00* | ***0.48±0.18*** | *0.21±0.02* |
| Δe4 | Initial content | **1.85±0.01** | 0.87±0.08 | 0.81±0.37 | 0.75±0.07 | *0.01±0.00* | ***0.61±0.13*** | *0.17±0.05* |
| | Uptake | **1.92±0.06** | 0.80±0.04 | 0.61±0.11 | 0.71±0.06 | *0.01±0.00* | ***0.46±0.03*** | *0.13±0.01* |
| | Chase | **1.95±0.07** | 0.85±0.06 | 0.45±0.08 | 0.69±0.01 | *0.01±0.00* | ***0.50±0.03*** | *0.16±0.03* |
| Δ*zupT* | Initial content | 0.78±0.01 | 0.95±0.03 | 0.61±0.02 | 0.74±0.03 | *0.01±0.00* | ***0.46±0.01*** | *0.32±0.12* |
| | Uptake | 0.77±0.03 | 0.79±0.04 | 0.46±0.06 | 0.68±0.03 | *0.01±0.00* | ***0.29±0.01*** | *0.27±0.09* |
| | Chase | 0.72±0.04 | 0.73±0.07 | 0.33±0.04 | ***0.62±0.04*** | *0.01±0.00* | ***0.25±0.02*** | *0.31±0.07* |
| Δ7 | Initial content | **2.66±0.09** | 0.86±0.05 | 2.84±0.73 | 0.71±0.01 | *0.01±0.00* | ***0.61±0.08*** | *0.40±0.05* |
| | Uptake | **2.62±0.12** | 0.84±0.03 | 3.43±0.38 | ***0.66±0.02*** | *0.01±0.00* | ***0.67±0.09*** | *0.31±0.04* |
| | Chase | **2.79±0.02** | 0.88±0.01 | 1.02±0.07 | 0.67±0.04 | *0.02±0.00* | 1.43±0.71 | *0.44±0.09* |
| Δ9 | Initial content | **2.38±0.20** | 0.85±0.04 | 3.25±1.29 | 0.76±0.07 | *0.02±0.00* | **1.63±0.57** | *0.28±0.07* |
| | Uptake | **2.52±0.18** | 0.94±0.03 | 3.39±1.17 | 0.77±0.06 | *0.01±0.00* | **1.88±0.75** | *0.26±0.07* |
| | Chase | **2.51±0.09** | 1.46±0.32 | 1.29±0.12 | 0.74±0.03 | *0.02±0.00* | **3.84±2.28** | *0.31±0.11* |

[a]This table shows the metal content of the metals other than zinc stemming from the $^{67}Zn$ pulse-chase experiments, the initial metal content, after the uptake period of 20 min and after the chase period of 40 min. Shown is the ratio compared to the metal content of strain AE104 cells grown in zinc-replete mZn medium. Bold and italics < 67%, Bold > 150%. The Mn values were close to the detection limit and not considered. The Co values from cells cultivated in lZn_lMg and lZn media just in italics. Grey field highlight an increased nickel content in Δ9 cells

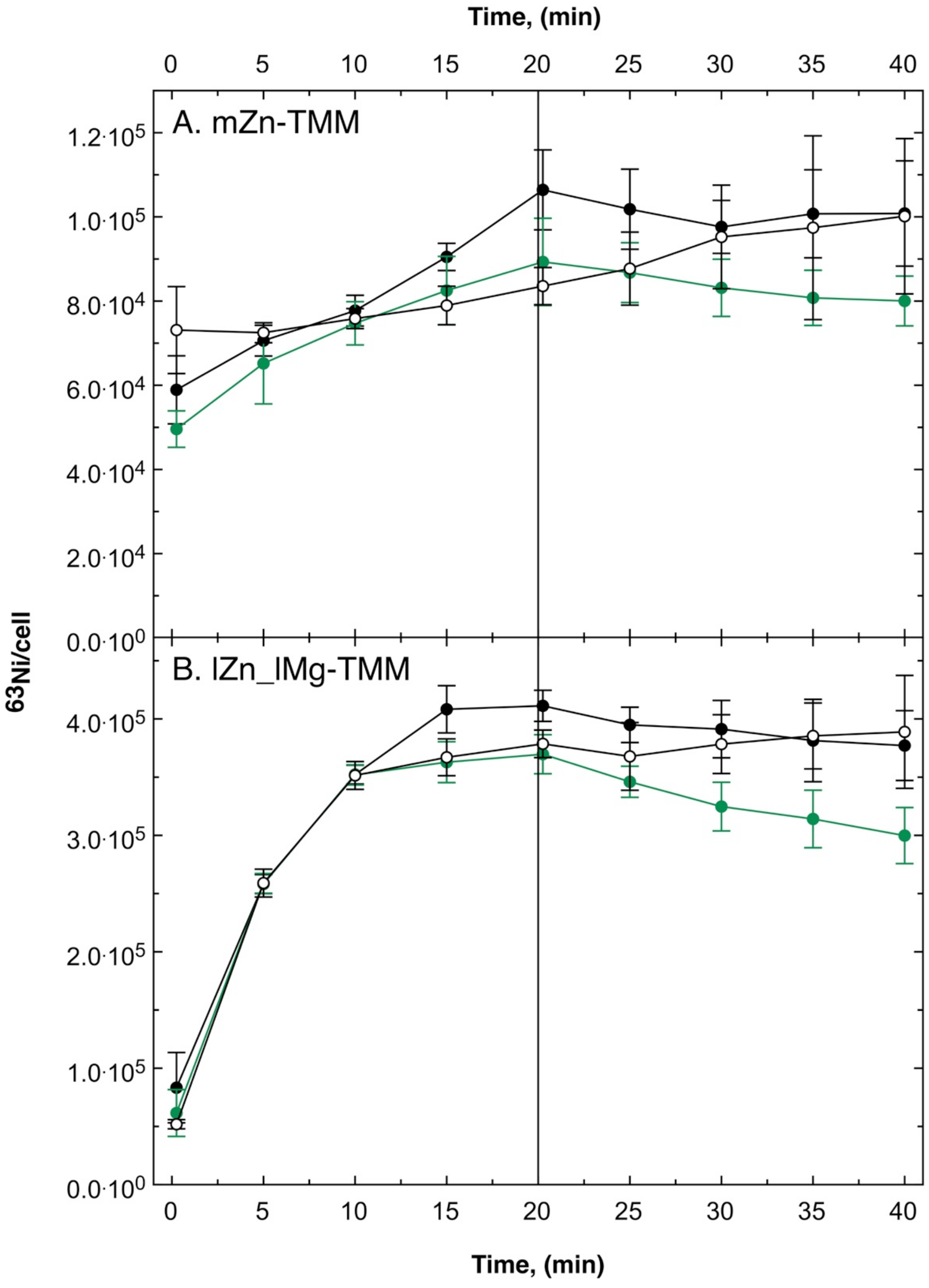


**Supplementary Figure S4. Pulse-chase-experiment with *C. metallidurans* strain AE104, nickel and zinc.** The cells were cultivated in TMM containing 200 nM Zn(II) (Panel A, mZn-TMM) or no added Zn(II) and 0.1 mM Mg(II) (Panel B, lZn_lMg, 35.2±30.4 nM Zn(II)) as described in Fig. 1. Pulse at t = 0 with 1 µM $^{63}$Ni(II), chase at t = 20 min with 100 µM non-radioactive Zn(II) (black circles), Ni(II) (green circles) or not chased (open circles)

**Supplementary Table S5. Accumulation of Mn, Co, Ni and Cd and pulse-chase after uptake with $^{63}$Ni.**

| | Uptake experiments[a] | | | |
|---|---|---|---|---|
| Medium | Mn | $10^3$ Co | $10^3$ Ni | $10^3$ Cd |
| mZn, 1 µM metal | 1443±904 | 18.97±2.48 | 4.64±0.92 | 27.9±1.6 |
| mZn, no addition | 746±288 | 7.37±1.05 | 3.39±0.20 | 0.13±0.09 |
| mZn, Q, (D) | 1.94, (0.58) | 2.57, (3.29) | 1.37, (1.12) | 220, (17) |
| lZn_lMg, 1 µM metal | 1527±167 | 80.86±2.90 | 8.25±0.17 | 54.3±0.2 |
| lZn_lMg, no addition | 260±92 | 0.22±0.11 | 5.85±0.35 | 0.07±0.04 |
| lZn_lMg, Q, (D) | 5.87, (4.90) | 372, (27) | 1.41, (4.66) | 733, (208) |
| | Pulse-chase experiments[b] | | | |
| Cells | mZn, $10^3$ Ni | mZn, $10^3$ Zn | lZn_lMg, $10^3$ Ni | lZn_lMg, $10^3$ Zn |
| Initial cells | 3.39±0.20 | 76.5±2.6 | 5.85±0.35 | 3.49±0.18 |
| 20 min 1 µM Ni(II) | 4.64±0.92 | 69.8±2.5 | 8.25±0.17 | 2.78±0.17 |
| Chase, 100 µM Zn(II) | 4.37±0.33 | 190±6 | 9.27±0.00 | 170±7 |
| Chase, 100 µM Ni(II) | 55.3±27.9 | 65.5±2.1 | 265±28 | 11.6±1.9 |

[a]In the uptake experiment, the cells were incubated for 20 min at 30°C with shaking with 1 µM of the indicated metal in mZn or lZn_lMg medium and the metal content was determined by ICP-MS. [b]Moreover, in a pulse-chase experiment, the cells were loaded with $^{63}$Ni instead of $^{65}$Zn and chased with 100 µM non-radioactive zinc or nickel. The results for three experiments with standard deviations are shown. Q gives the ratios with/without the added metal ions, D the respective distance value of this comparison.

## Role of uptake systems.

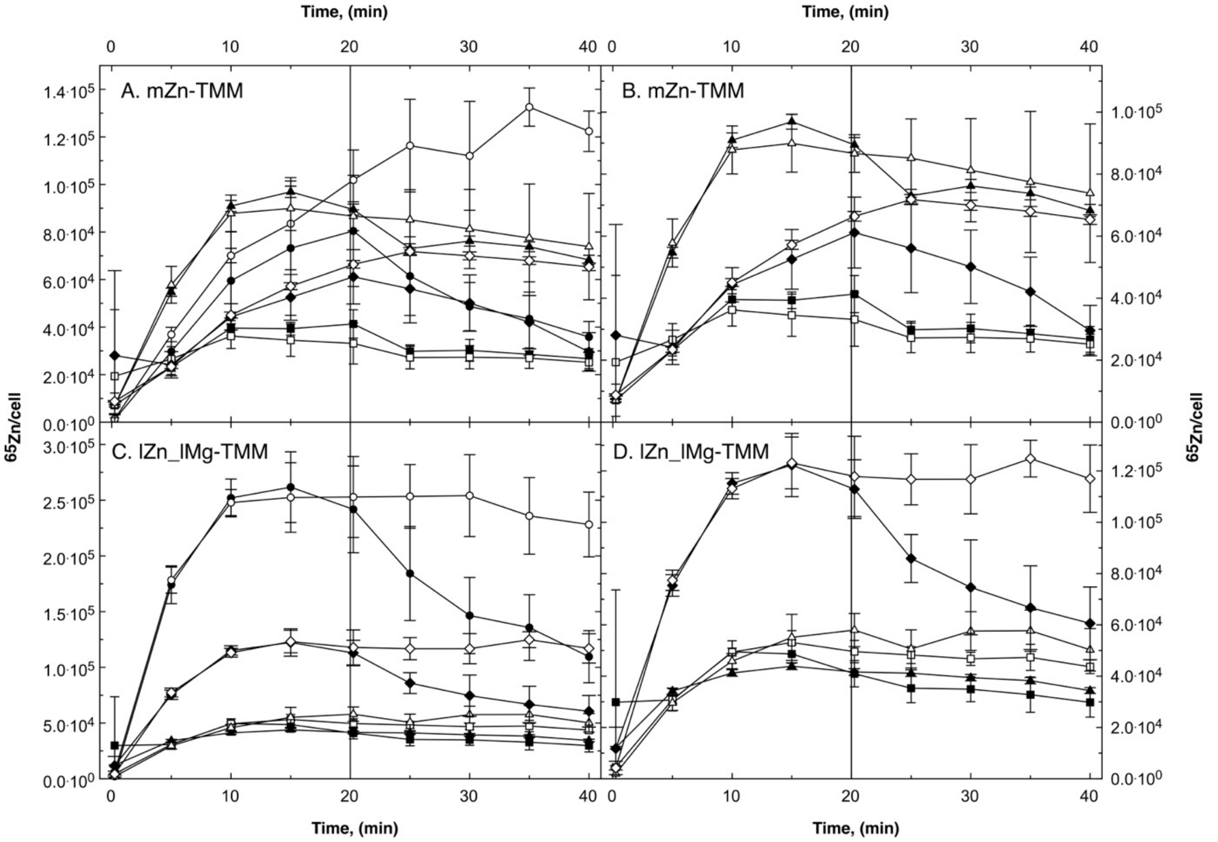


**Supplementary Figure S5. Pulse-chase-experiment with *C. metallidurans* strains AE104 and uptake mutants Δ*zupT*, Δ7 and Δ9.** Cells of strain AE104 (circles), Δ*zupT* (diamonds), Δ7 (Δ*zupT* Δ*corA1* Δ*corA2* Δ*corA3* Δ*zntB* Δ*pitA* Δ*hoxN,* squares) and Δ9 (Δ7 Δ*mgtA* Δ*mgtB::kan,* triangles) were cultivated in TMM containing 200 nM Zn(II) (mZn-TMM, Panels A and B) or no added Zn(II) and 0.1 mM Mg(II) (lZn_lMg, Panels C and D) as described in Fig. 2. Pulse at t = 0 with 1 µM $^{65}$Zn(II), chase at t = 20 min with 100 µM non-radioactive Zn(II) (black symbols), or not chased (open symbols). Panels B and D contain the same data as Panels A and C with the exception of the AE104 values.

**Role of efflux systems.**

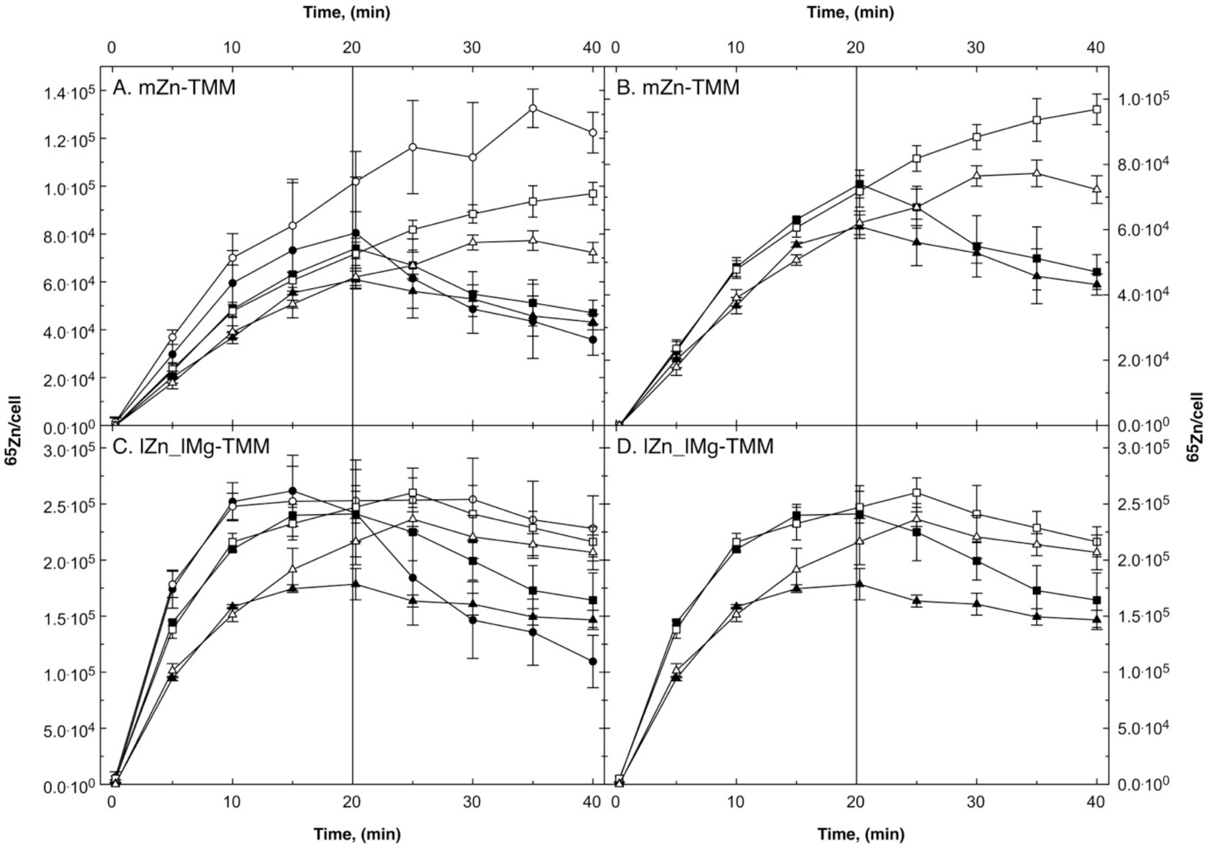


**Supplementary Figure S6. Pulse-chase-experiment with *C. metallidurans* strains AE104 and efflux mutants ∆e2 and ∆e4.** Cells of strain AE104 (circles), ∆e2 (∆*zntA* ∆*cadA,* squares) and ∆e4 (∆e2 ∆*dmeF* ∆*fieF,* triangles) were cultivated in TMM containing 200 nM Zn(II) (mZn-TMM, Panels A and B) or no added Zn(II) and 0.1 mM Mg(II) (lZn_lMg, Panels C and D) as described in Fig. 2. Pulse at t = 0 with 1 µM $^{65}$Zn(II), chase at t = 20 min with 100 µM non-radioactive Zn(II) (black symbols), or not chased (open symbols). Panels B and D contain the same data as Panels A and C with the exception of the AE104 value

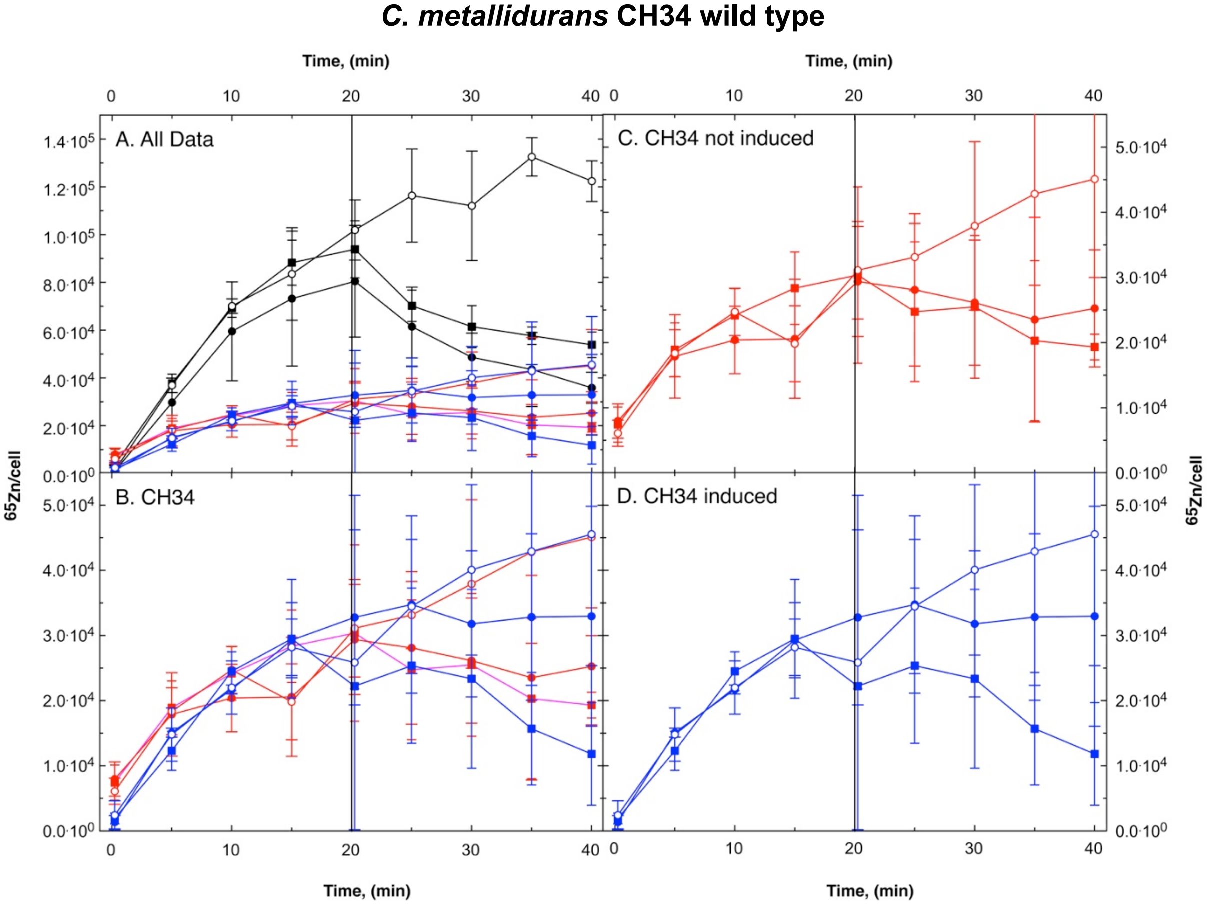


**Supplementary Figure S7. Pulse-chase experiment with *C. metallidurans* strains AE104 and CH34 wild type.** Cells of strain AE104 (black) and CH34 (red) were cultivated in mZn-TMM containing 200 nM Zn(II) or 100 µM Zn(II) to induce the *czc* metal resistance determinant on plasmid pMOL30 in strain CH34 (blue) as described in Fig. 2. Pulse at t = 0 with 1 µM $^{65}$Zn(II), chase at t = 20 min with 100 µM non-radioactive Zn(II) (closed circles), 1 mM non-radioactive Zn(II) (closed squared) or not chased (open circles). Panel A contains all CH34 data and those from strain AE104 for comparison, Panel B only the CH34 data, Panel C the data from not-induced CH34 cells and Panel D those from induced CH34 cells.

## Literature of the Supplement


1. Mergeay M, Nies D, Schlegel HG, Gerits J, Charles P, van Gijsegem F. 1985. *Alcaligenes eutrophus* CH34 is a facultative chemolithotroph with plasmid-bound resistance to heavy metals. J Bacteriol 162:328-334.
2. Hirth N, Gerlach MS, Wiesemann N, Herzberg M, Grosse C, Nies DH. 2023. Full copper resistance in *Cupriavidus metallidurans* requires the interplay of many resistance systems. Appl Environ Microbiol 89:10.1128/aem.00567-23.
3. Scherer J, Nies DH. 2009. CzcP is a novel efflux system contributing to transition metal resistance in *Cupriavidus metallidurans* CH34. Mol Microbiol 73:601-621.
4. Kirsten A, Herzberg M, Voigt A, Seravalli J, Grass G, Scherer J, Nies DH. 2011. Contributions of five secondary metal uptake systems to metal homeostasis of *Cupriavidus metallidurans* CH34. J Bacteriol 193:4652-4663.
5. Herzberg M, Bauer L, Kirsten A, Nies DH. 2016. Interplay between seven secondary metal transport systems is required for full metal resistance of *Cupriavidus metallidurans*. Metallomics 8:313-326.
6. Grosse C, Herzberg M, Schüttau M, Nies DH. 2016. Characterization of the $\Delta 7$ mutant of *Cupriavidus metallidurans* with deletions of seven secondary metal uptake systems. mSystems 1:e00004-16.
7. Legatzki A, Franke S, Lucke S, Hoffmann T, Anton A, Neumann D, Nies DH. 2003. First step towards a quantitative model describing Czc-mediated heavy metal resistance in *Ralstonia metallidurans*. Biodegradation 14:153-168.
